# The EDA-deficient mouse has Zymbal's gland hypoplasia and acute otitis externa

**DOI:** 10.1242/dmm.049034

**Published:** 2022-03-30

**Authors:** Jorge del-Pozo, Denis J. Headon, James D. Glover, Ali Azar, Sonia Schuepbach-Mallepell, Mahmood F. Bhutta, Jon Riddell, Scott Maxwell, Elspeth Milne, Pascal Schneider, Michael Cheeseman

**Affiliations:** 1 Veterinary Pathology, The Royal (Dick) School of Veterinary Studies, University of Edinburgh, Edinburgh EH25 9RG, UK; 2 Roslin Institute and The Royal (Dick) School of Veterinary Studies, University of Edinburgh, Edinburgh EH25 9RG, UK; 3 Department of Biochemistry, University of Lausanne, Boveresses 155, CH-1066 Epalinges, Switzerland; 4 Department of ENT, Royal Sussex County Hospital, Brighton BN2 5BE, UK; 5 Brighton and Sussex Medical School, Falmer, Brighton BN1 9PX, UK; 6 Division of Pathology, University of Edinburgh, Institute of Genetics & Molecular Medicine, Crewe Road, Edinburgh EH4 2XR, UK; 7 Centre for Comparative Pathology, Division of Pathology, University of Edinburgh, Institute of Genetics & Molecular Medicine, Crewe Road, Edinburgh EH4 2XR, UK

**Keywords:** Hypohidrotic ectodermal dysplasia, Sparse and wavy hair rat, EDARADD, FBXO11, MECOM, EDAR, Tabby mouse

## Abstract

In mice, rats, dogs and humans, the growth and function of sebaceous glands and eyelid Meibomian glands depend on the ectodysplasin signalling pathway. Mutation of genes encoding the ligand EDA, its transmembrane receptor EDAR and the intracellular signal transducer EDARADD leads to hypohidrotic ectodermal dysplasia, characterised by impaired development of teeth and hair, as well as cutaneous glands. The rodent ear canal has a large auditory sebaceous gland, the Zymbal’s gland, the function of which in the health of the ear canal has not been determined. We report that EDA-deficient mice, EDAR-deficient mice and EDARADD-deficient rats have Zymbal’s gland hypoplasia. *Eda^Ta^* mice have 25% prevalence of otitis externa at postnatal day 21 and treatment with agonist anti-EDAR antibodies rescues Zymbal’s glands. The aetiopathogenesis of otitis externa involves infection with Gram-positive cocci, and dosing pregnant and lactating *Eda^Ta^* females and pups with enrofloxacin reduces the prevalence of otitis externa. We infer that the deficit of sebum is the principal factor in predisposition to bacterial infection, and the *Eda^Ta^* mouse is a potentially useful microbial challenge model for human acute otitis externa.

## Abstract (precis)

**Summary:** Ectodysplasin-deficient mice have growth retardation of the auditory sebaceous Zymbal's gland and are predisposed to spontaneous bacterial infection of the outer ear canal by opportunistic pathogens.

## INTRODUCTION

Human acute otitis externa (AOE) is a common microbial infection of the ear canal (external acoustic meatus), with an estimated 1.72 million cases in the US in 2014 and an estimated annual treatment cost of $564 million (Collier et al., 2021). The ear canal is the only cul-de-sac keratinising skin surface in the body and is self-cleansing through the production of cerumen (wax), which is a mixture of desquamated keratinocytes and glandular secretions (Guest et al., 2004). The human ear canal has sebaceous glands associated with hair follicles (pilosebaceous units) that produce lipid-rich sebum (Bortz et al., 1990; Guest et al., 2004), and ceruminous glands, which are modified apocrine glands that secrete fluid rich in antibacterial peptides (Main and Lim, 1976; Stoeckelhuber et al., 2006). Wetting of the ear canal through swimming, bathing or high environmental humidity compromises these defences and predisposes the canal to microbial infection, giving rise to its common name ‘swimmer's ear’.

Animal models of AOE generally involve disrupting the ear canal epithelial barrier by mechanical abrasion, sustained wetting or chemical irritants and inoculation of a microbial pathogen. Animal models include rats (Emgård and Hellström, 1997, 2001; Emgård et al., 2005; Demirel et al., 2018), guinea pigs (Wright and Dineen, 1972; King and Estrem, 1990; Zhai et al., 2014) and mice (Wright et al., 2000). However, spontaneous otitis externa is not a notable disease of laboratory rats, guinea pigs or mice in lab animal medicine texts (Fox et al., 2015).

Rodents lack the apocrine ceruminous glands that are found in human, dog, goat and pig ear canals (Wang et al., 2021), but have a specialised large multilobulated auditory sebaceous gland, also known as the Zymbal's gland (Rudmann et al., 2012), which opens via a duct into the ear canal close to the tympanic membrane. The Zymbal's gland is also called the ear-wax gland (glandula ceruminosa) (Grüneberg, 1971) or ceruminous gland (Berry et al., 1994; Mozaffari et al., 2021). Hereafter, we use the term Zymbal's gland to draw a distinction between this specialised sebaceous holocrine gland and the human apocrine ceruminous gland. The ear canal carries sound waves received by the outer ear, the pinna, towards the tympanic membrane, and in the mouse the canal is formed by a short osseous part, the ectotympanic ring, and a short annular cartilage (Navarro et al., 2017). Vibrations of the tympanic membrane are transmitted through the air-filled auditory bulla (middle ear) to the inner ear by the ossicular chain.

Sebaceous glands develop either as paired outgrowths of the hair follicle or in specialised glands, such as the Meibomian, preputial, clitoral and Zymbal's glands, which develop independently of the hair follicle (Dhouailly and Oftedal, 2016). Preputial glands develop at embryonic day (E)14.5 as placodes in the epidermis of the genital tubercle, and the Meibomian gland placodes develop at the fused eyelid margins at E18.5. The embryological development of the Zymbal's gland is less well studied (Dhouailly and Oftedal, 2016) but the primordium is reported to be present at E15 (Grüneberg, 1971).

The growth and function of hair follicle sebaceous glands and Meibomian glands are dependent on the ectodysplasin signalling pathway, which comprises a TNF-like ligand ectodysplasin (EDA), its transmembrane receptor EDAR and the intracellular signal transducer EDARADD. The loss of signalling due to mutation of genes in this linear pathway leads to hypohidrotic ectodermal dysplasia (HED) and impairs the development of teeth and hair, as well as cutaneous glands (Kowalczyk-Quintas and Schneider, 2014). The *Tabby* (*Eda^Ta^*) mouse is a model of X-linked HED (XLHED) and is deficient in EDA. Treatment of adult *Eda^Ta^* mice with agonist anti-EDAR antibody (Kowalczyk et al., 2011; Kowalczyk-Quintas et al., 2014) restores sebaceous gland growth and sebum production, and sustained treatment of *Eda^Ta^* and wild-type mice heightens sebum production (Kowalczyk-Quintas et al., 2015). In addition, prenatal correction of XLHED with a recombinant protein that includes the receptor binding domain of EDA restores Meibomian gland growth in humans (Schneider et al., 2018) and dogs (Margolis et al., 2019). Adult mouse sebaceous glands express the *EDAR* gene, suggesting the action of the agonist anti-EDAR antibody treatment is directly on the sebaceous glands (Kowalczyk-Quintas et al., 2015). The rat Zymbal's gland expresses the *EDARADD* gene (del-Pozo et al., 2019a), so it is also likely to be stimulated directly by EDAR signalling.

Growth retardation of Meibomian glands causes dry eye, keratitis, ulceration and corneal neovascularization in *Eda^Ta^* mice. The aetiopathogenesis is related to loss of lipid secretion altering tear film stability, a reduction in goblet cell density and increased susceptibility to eyelid and conjunctival inflammation through loss of EDA function (Cui et al., 2005; Wang et al., 2016). Furthermore, the Meibomian gland secretes EDA, and this is important for corneal epithelial homeostasis (Li et al., 2017). EDA signalling is also important for lacrimal gland development and function, and also contributes to corneal homeostasis and repair (Kuony et al., 2019).

The Zymbal's gland in the rat and mouse is known principally from toxicology studies as a target for chemical carcinogens (Gold et al., 2001; Rudmann et al., 2012), and its normal physiological role is not documented. Postnatal day (P)10 *Eda^Ta^* mice have Zymbal's glands that are ∼5% of normal size and fewer ear canal hair follicles, which have smaller sebaceous glands than wild-type mice (Grüneberg, 1971), but the functional consequences of these deficits have not been investigated. In this study, we have investigated Zymbal's gland growth and the health of the ear canal in the *Eda^Ta^* mouse and in the EDARADD-deficient short and wavy hair rat (*Edaradd^swh/swh^*) strain (Kuramoto et al., 2005, 2011). The *Eda^Ta^* mouse and the *Edaradd^swh/swh^* rat have deficits in the nasopharyngeal submucosal glands, which protect the auditory (eustachian) tube, and this predisposes them to otitis media, infection and inflammation of the auditory bulla. Prenatal treatment of *Eda^Ta^* mice with agonist anti-EDAR antibody rescues the submucosal glands and prevents otitis media (del-Pozo et al., 2019a).

The human tympanic membrane can perforate in recurrent acute otitis media (Folino et al., 2021) and in acute and chronic suppurative otitis media (Mozaffari et al., 2020). Drainage of mucopurulent exudate though a tympanic membrane perforation (otorrhea) can result in the infection of the ear canal and AOE (Rosenfeld et al., 2014). Otitis externa is common in dogs, accounting for 7-10% of all canine cases seen in veterinary practice (O'Neill et al., 2014, 2021), and tympanic membrane perforation/otitis media is considered to be a common perpetuating cause of chronic otitis externa (Saridomichelakis, et al., 2007; Lorek et al., 2020). To evaluate the potential impact of otitis media on the tympanic membrane, and on the health of ear canal, we included two additional mouse models of chronic otitis media as an outgroup comparison. The aetiopathogeneses of otitis media in the *Fbxo11^Jf/+^* mouse is associated with a bulla cavitation defect (del-Pozo et al., 2019b), and the *Mecom^Jbo/+^* mouse has dysregulated NFkB inflammatory responses (Xu et al., 2012).

We found that *Eda^Ta^* and *Edar^OVE1B/OVE1B^* mice, and *Edaradd^swh/swh^* rats, have Zymbal's gland hypoplasia. In addition, the *Eda^Ta^* mouse has hypoplasia of ear canal pilosebaceous units (hypotrichosis) and is predisposed to bacterial otitis externa. The Zymbal's glands and ear canal pilosebaceous units in P21 *Eda^Ta^* mice are rescued by treatment with agonist anti-EDAR antibody. Furthermore, treatment with the broad-spectrum antibiotic enrofloxacin reduces the prevalence of otitis externa in P21 *Eda^Ta^* mice.

## RESULTS

### Zymbal's gland hypoplasia in *Eda^Ta^* mice

**Fig. 1.**
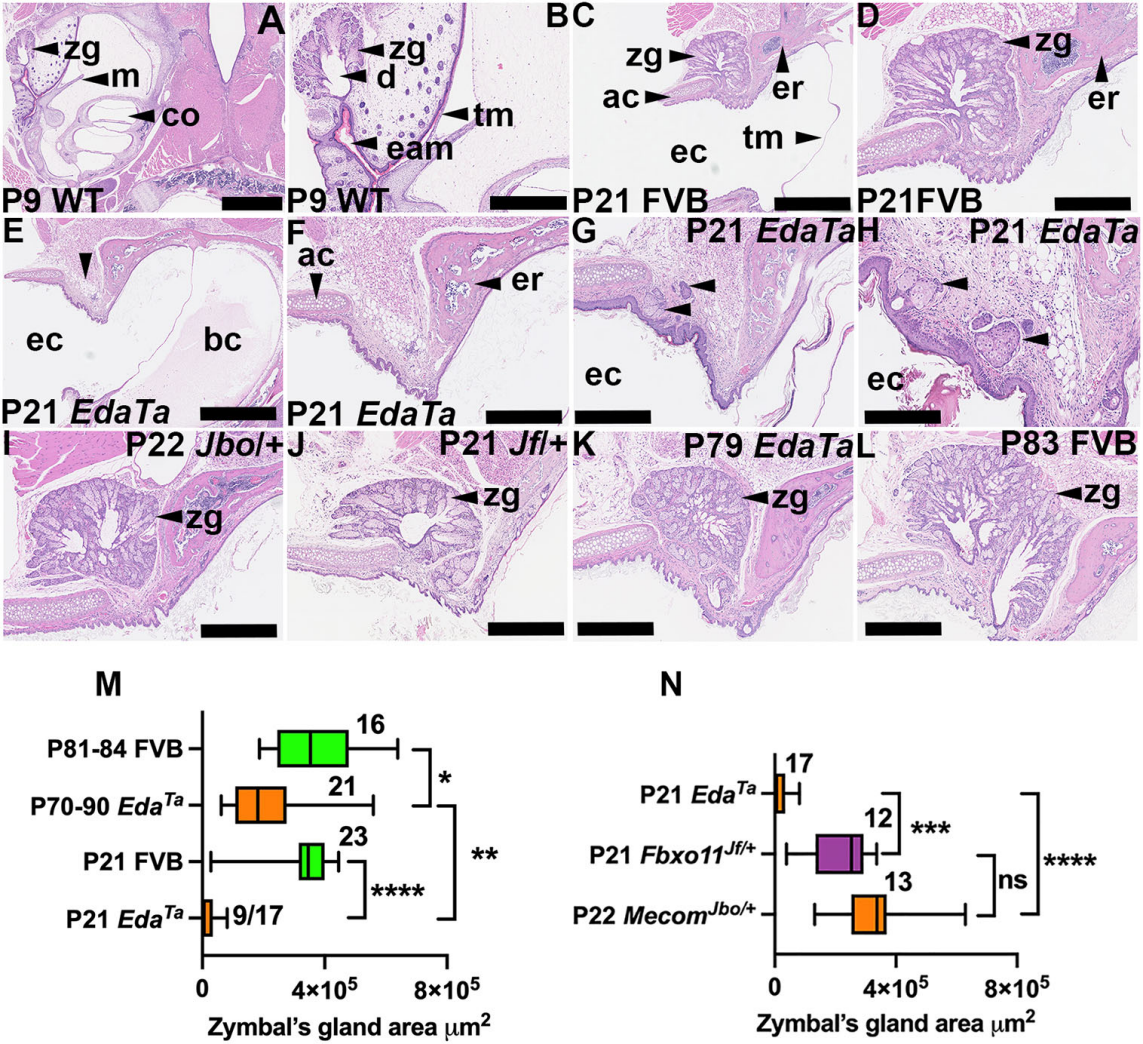
**P21 *Eda^Ta^* mice have Zymbal's gland growth retardation and hypoplasia.** Dorsal plane H&E-stained sections through the bulla and outer ear canal. The image upper margin is rostral, and the left is lateral. A and B, C and D, and E and F, are paired low and high magnification images of the same section. (A,B) P9 wild-type (mixed C57BL/6J C3H/HeH genetic background) Zymbal's gland has a widely patent duct and a narrowly canalised external auditory meatus (ear canal) lined with keratinised epithelium. (C,D) P21 FVB Zymbal's gland located in the rostral ear canal wall between the ectotympanic ring and annular cartilage. (E-H) P21 *Eda^Ta^* mice. (E,F) Zymbal's gland is not present in its normal location (vertical arrowheads). (G,H) Examples of small Zymbal's glands in the dermis, and those associated with the skin surface lack hair follicles. (I,J) P22 *Mecom^Jbo/+^* (I) and P21 *Fbxo11^Jf/+^* (J) mutants that, like *Eda^Ta^* mice, are otitis media prone strains but have normal-sized Zymbal's glands. (K,L) By P79, growth of the Zymbal's gland in *Eda^Ta^* mice has increased (K) but has not achieved the size of P83 FVB control mice (L). (M,N) Morphometric analysis of Zymbal's gland size. (M) Zymbal's gland size is reduced in P21 and P79-P90 *Eda^Ta^* mice compared to control FVB mice. (N) Zymbal's gland size is reduced in P21 *Eda^Ta^* compared to P21 *Fbxo11^Jf/+^* and P22 *Mecom^Jbo/+^* mice. The fraction adjacent to the data is the number of ears in which the Zymbal's gland is present out of the total number examined. Data in graphs are presented as Tukey's box-and-whisker plots (sample numbers adjacent to box). Boxes represent the 25% percentile, the median and the 75% percentile, and the whiskers represent the minimum and the maximum. *P<0.05; **P<0.01; ***P<0.001; ****P<0.0001; ns, not significant (two-tailed Kruskal–Wallis test followed by Dunn's multiple comparison test). ac, annular cartilage; bc, bulla cavity; co, cochlea; d, duct; eam, external auditory meatus; ec, ear canal; er, ectotympanic ring; m, malleus; tm, tympanic membrane; zg, Zymbal's gland. Scale bars: 1000 µm (A,C,E); 500 µm (B,D,F,G,I-L); 250 µm (H).

The mouse external auditory meatus opens and extends on E12.5 (Mallo et al., 2000; Minoux et al., 2013), then closes between E15-P7 before re-opening from P7 to P12, and is fully opened at P12 (Anthwal and Thompson, 2016). We observed that the P9 Zymbal's gland has a widely patent duct that opens into a narrowly canalised external auditory meatus (Fig. 1A,B).

The Zymbal's gland is located between the ectotympanic ring and the annular cartilage on the rostral surface of the ear canal, and we measured *Eda^Ta^* Zymbal's tissue in this defined region. Hair follicles are reduced in number in *Eda^Ta^* ear canal skin (Grüneberg, 1971) but we were careful to exclude any sebaceous glands associated with a hair follicle. Examination of serial step sections did not reveal Zymbal's glands in 8 of 17 P21 *Eda^Ta^* ear canals (Fig. 1E,F) but those detected were ∼8% the size of P21 FVB mouse controls (Fig. 1C,D,G,H,M). Depending on the plane of section, *Eda^Ta^* Zymbal's glands appeared as variably sized sebocyte lobules (Fig. 1G,H) and ducts connecting with the skin surface (Fig. S4P). Sebocyte lobules in P21 *Eda^Ta^* Zymbal's glands were significantly larger (median 10,494 µm^2^, 95% c.i. 3584–17,786 µm^2^, *n*=32) than P21 FVB hair follicle sebaceous glands (median 1273 µm^2,^ 95% c.i. 1034-1747 µm^2^, *n*=33; *P*<0.0001, Mann–Whitney test). In contrast to P21 mice, all P79-P90 *Eda^Ta^* had histologically unremarkable Zymbal's glands, and although these were significantly larger than at P21, they were only ∼50% the size of Zymbal's glands in P81-P84 FVB mice (Fig. 1K-M).

*Eda^Ta^*, *Mecom^Jbo/+^* and *Fbxo11^Jf/+^* mutants all have middle ear pathology but its importance in the development of ear canal disease is unknown. To help differentiate the contributions of middle ear pathology and reduced Zymbal's gland size in *Eda^Ta^* mice, we compared gland size between mutant strains and found P22 *Mecom^Jbo/+^* and P21 *Fbxo11^Jf/+^* mice have larger Zymbal's glands than in P21 *Eda^Ta^* mice (Fig. 1I,J,N).

### Otitis externa in P21 *Eda^Ta^* mice

**Fig. 2.**
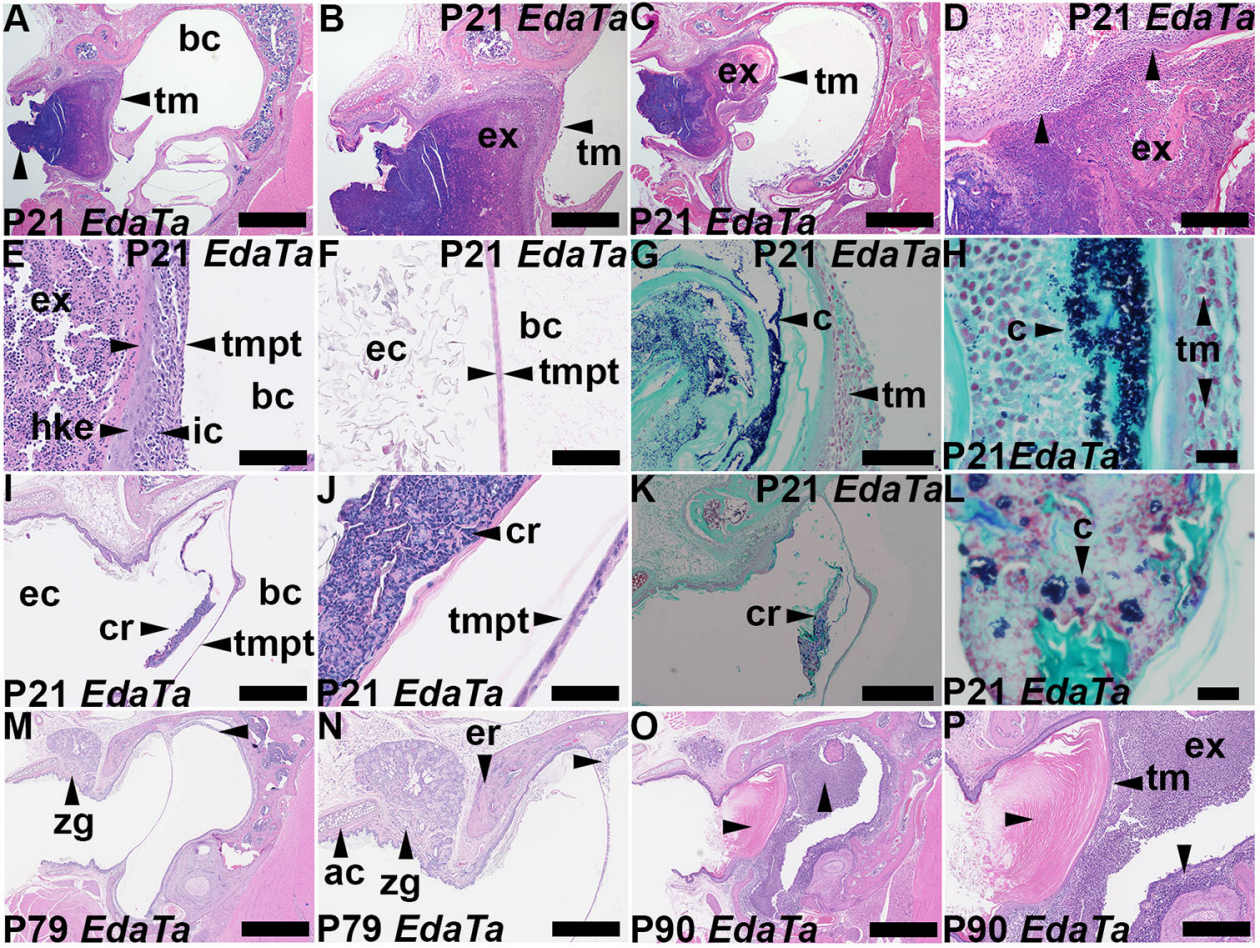
**P21 *Eda^Ta^* mice have otitis externa.** Dorsal plane H&E- and Gram-stained sections through the bulla and ear canal of *Eda^Ta^* mice. The image upper margin is rostral, and the left is lateral. A and B, C and D, E and F, I and J, M and N, and O and P are paired low and high magnification images of the same section. (A-E) Otitis externa. The tympanic membrane is intact and the ear canal contains a plug of exudate comprising neutrophils mixed with squamous cells. (D) Ear canal dermal and epithelial inflammation (arrowheads mark the margins of epithelial erosion). (E) Thickened inflamed tympanic membrane pars tensa (arrowheads) with exudate on its external surface. Hyperplastic keratinising epithelium and inflammatory cell infiltration of the lamina propria can be observed. (F) Normal tympanic membrane in an unaffected ear. (G,H) Examples of ear canal exudate with Gram-positive cocci (Gram Twort stain). (I-L) Other P21 *Eda^Ta^* mice have thin crusts overlying the tympanic membrane containing neutrophils and Gram-positive cocci (K,L). There is no marked inflammatory reaction in the skin of the ear canal. (M-P) P79 (M,N) and P90 *Eda^Ta^* (O,P) mice. (M,O) The Zymbal's gland is present and there is no otitis externa. (M,N) Minimal intrabullar exudate (otitis media; horizontal arrowheads). (O,P) The ear canal contains a plug of bland squamous cells. Severe otitis media with neutrophilic exudate in the bulla cavity and thickened inflamed mucosa can be observed (vertical arrowheads). ac, annular cartilage; bc, bulla cavity; c, Gram-positive cocci; cr, crust; d, duct; ec, ear canal; er, ectotympanic ring; ex, inflammatory exudate; hke, hyperplastic keratinising epithelium; ic, inflammatory cell infiltrate; tm, tympanic membrane; tmpt, tympanic membrane pars tensa; zg, Zymbal's gland. Scale bars: 1000 µm (A,C,I,M,O); 500 µm (B,I,K,N); 200 µm (D,P); 100 µm (E-G); 50 µm (J); 20 µm (H,L).

Six of twelve P21 *Eda^Ta^* mice had unilateral otitis externa (see Materials and Methods for diagnostic criterion) (Fig. 2A-H). These cases occurred in two litters of five and seven pups born to different parents; its prevalence of 25% (6 of 24 ears) was significantly elevated compared with the control group of P21 FVB mice in which otitis externa was absent (*n*=26 ears; *P*=0.0085, Fisher's Exact test). In all cases of otitis externa, the neutrophil-rich suppurative exudates contained Gram-positive cocci (Fig. 2G,H), but no fungi were detected using either PAS or Grocott stains.

Otitis externa was not observed in P79-90 *Eda^Ta^* (*n*=52 ears) (Fig. 2M-P) or in P81-P84 FVB (*n*=20 ears). The ear canal of some P79-P90 *Eda^Ta^* mice can contain a thick plug of bland squamous epithelial cells that lack an inflammatory component (Fig. 2O,P).

The occurrence of otitis externa was assessed in *Mecom^Jbo/+^* and *Fbxo11^Jf/+^* mice. Neither mutants or wild-type littermates had otitis externa at weaning age or as older adults [*Mecom^Jbo/+^* (P22, *n*=14; P84, *n*=12 ears); *Mecom*^+*/+*^ (P22, *n*=14 ears); *Fbxo11^Jf/+^* (P21, *n*=13; P57-P223, *n*=41 ears); *Fbxo11*^+*/+*^ mice (P21, *n*=10 ears)].

Thirty-eight percent (6 of 16) of P21 *Eda^Ta^* ears unaffected by otitis externa had a thin crust of desquamated epithelial cells, neutrophils and Gram-positive cocci on the external surface of the tympanic membrane (Fig. 2I-L); these crusts were <10% of the size of otitis externa exudate accumulations (Fig. S1A), and there was no accompanying inflammatory thickening of the tympanic membrane (Fig. 2I,J). Ears with tympanic membrane crusts had thickened ear canal soft tissue (Fig. S1B) but dermal and intra-epithelial neutrophil infiltration were also absent. Tympanic membrane crusts comprising desquamated cells and neutrophils (but not bacteria) were found in 21% (3 of 14) of P22 *Mecom^Jbo/+^* ears but were absent in P22 *Fbxo11^Jf/+^* (*n*=10) and wild-type control ears (P21 FVB, *n*=26; P22 *Mecom*^+*/+*^, *n*=14; P21 *Fbxo11*^+*/+*^, *n*=14).

### Ear canal hypotrichosis in *Eda^Ta^* mice

**Fig. 3.**
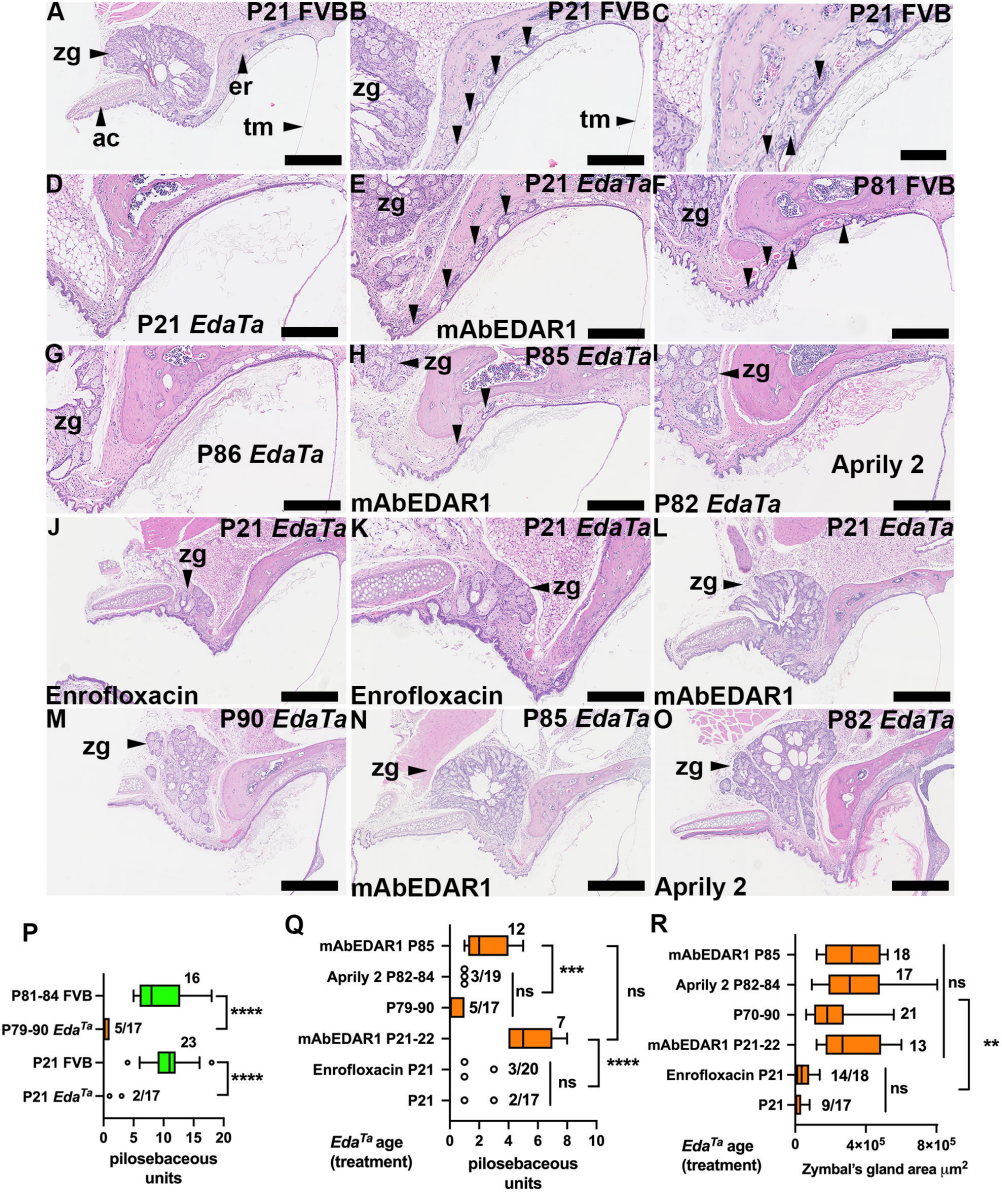
**Partial restoration of ear canal pilosebaceous unit density and Zymbal's gland growth by treatment of *Eda^Ta^* mice with agonist anti-EDAR antibodies.** (A-O) Dorsal plane sections of ear canal skin in between the tympanic membrane and the annular cartilage in *Eda^Ta^* and FVB mice. Panel orientation: left is lateral, right medial, top rostral and bottom caudal. Panel groups A, B and C, and J and K, are different magnifications of the same section. Pilosebaceous units are indicated by unlabelled arrowheads. (A-C) P21 FVB have normal Zymbal's glands and multiple hair follicles with associated sebaceous glands (pilosebaceous units). (D,E) Hypotrichosis in P21 *Eda^Ta^* ear canal (D) and restoration of pilosebaceous units (and Zymbal's gland) with agonist anti-EDAR antibody (mAbEDAR1) administered prenatally and postnatally (E). (F) Normal P81 FVB. (G) Hypotrichosis in an untreated P86 *Eda^Ta^* mouse. (H) Partial restoration of pilosebaceous units in P85 *Eda^Ta^* mouse with agonist anti-EDAR antibody administered prenatally, but not with treatment with isotype control antibody (Aprily 2) administered prenatally (I). (J-O) Zymbal's gland. (J,K) Enrofloxacin treatment of P21 *Eda^Ta^* mice does not impact on small Zymbal's gland size. Panel K is a higher magnification image of J. (L) Restoration of Zymbal's gland with agonist anti-EDAR antibody administered prenatally. (M) Zymbal's gland growth occurs independently of EDA-EDAR signalling in P90 *Eda^Ta^* mice. (N,O) Prenatal agonist anti-EDAR antibody mAbEDAR1 (N) or isotype antibody (Aprily 2) (O) do not enhance Zymbal's gland growth more than in untreated mice. (P,Q) Morphometric analysis of pilosebaceous unit density in the ear canal. (P) FVB mice have a higher density of pilosebaceous units than *Eda^Ta^* mice at P21 and >P79. (Q) Partial restoration of pilosebaceous unit density with prenatal and postnatal administration of agonist anti-EDAR antibody in P21 and P85 *Eda^Ta^* mice, but not with isotype antibody at P82-P84. Enrofloxacin treatment does not impact on *Eda^Ta^* ear canal pilosebaceous unit density at P21. (R) Partial restoration of Zymbal's gland size with prenatal and postnatal administration of agonist anti-EDAR antibody in P21 *Eda^Ta^* mice. Prenatal and postnatal agonist anti-EDAR antibody mAbEDAR1 or isotype antibody (Aprily 2) do not enhance Zymbal's gland growth more than in untreated mice at P79-P90. Enrofloxacin treatment does not impact on *Eda^Ta^* Zymbal's gland size at P21. Zymbal's glands and pilosebaceous unit measurements could not be made in all ear specimens (see Materials and Methods for exclusion criteria), and the number of ears assessed for each feature can be different. Furthermore, Zymbal's gland and pilosebaceous units were not detected in all *Eda^Ta^* ears. In panels P-R, the fraction adjacent to the data is the number of ears in which the feature is present out of the total number examined. Data in graphs are presented as Tukey's box-and-whisker plots (sample numbers adjacent to box, and the points are outliers). Boxes represent the 25% percentile, the median and the 75% percentile, and the whiskers represent the minimum and the maximum. Data for untreated P21 and P70-P90 *Eda^Ta^* are from Fig. 1. **P<0.01; ***P<0.001; ****P<0.0001; ns, not significant (two-tailed Kruskal–Wallis test followed by Dunn's multiple comparison test). ac, annular cartilage; er, ectotympanic ring; tm, tympanic membrane pars tensa; zg, Zymbal's gland. Scale bars: 500 µm (A,J,L-O); 250 µm (B,D-I,K); 100 µm (C).

The skin of the osseous ear canal was haired in P21 and P81-P84 FVB mice (Fig. 3A-C,F), but was sparsely haired in P21 and P79-P90 *Eda^Ta^* mice (Fig. 3D,G,P). The skin over the annular cartilage was thin and sparsely haired (Fig. 3A,J,K). No apocrine glands were found in the osseous or cartilaginous regions of the ear canal in mice P21 FVB mice (*n*=6).

### Partial restoration of Zymbal's gland growth and ear canal pilosebaceous unit density in *Eda^Ta^* mice with agonist anti-EDAR antibody treatment

*Eda^Ta^* mice treated prenatally or prenatally and postnatally with agonist anti-EDAR antibody had a higher density of ear canal pilosebaceous units at P21 (Fig. 3D,E,Q) and larger Zymbal's glands than untreated (Fig. 3L,R) or antibiotic (enrofloxacin) mice (Fig. 3J,K). Prenatal agonist anti-EDAR antibody treatment rescued ear canal pilosebaceous units in P85 *Eda^Ta^* mice (Fig. 3H,Q). However, post-weaning growth of Zymbal's glands continued independently of EDA-EDAR signalling, and gland size in mice treated prenatally with agonist anti-EDAR antibody was no greater in P85 *Eda^Ta^* mice than in untreated P79-P90 mice or P82-P84 mice treated with isotype antibody control (Fig. 3M-O). Furthermore, the density of ear canal pilosebaceous units was low in isotype antibody-treated P82-P84 *Eda^Ta^* mice (Fig. 3I,Q).

### Association between otitis externa susceptibility and small Zymbal's gland size

Otitis externa and tympanic membrane crusts were not observed in agonist anti-EDAR treated P21 *Eda^Ta^* mice (*n*=8). In contingency tests, the prevalence of otitis externa in treated mice was statistically lower than in untreated P21 controls (*n*=12, Table S1, Fig. S2A). Furthermore, small Zymbal's gland size in weaning-aged mice was strongly associated with otitis externa [six of 12 affected P21 mice versus 0 of 45 P79-P90 *Eda^Ta^* mice (untreated, and those administered agonist anti-EDAR or isotype antibodies); *P*=0.0000255, Fisher's Exact test].

### Co-existence of otitis media and otitis externa in P21 *Eda^Ta^* ears

To gauge the importance of otitis media in the development of otitis externa at P21 and P22, we made an outgroup comparison and found that the prevalence of otitis media was higher in otitis media-sensitive, but otitis externa-resistant, mouse strains *Mecom^Jbo/+^* and *Fbxo11^Jf/+^* mice (13 of 14, and 9 of 10 ears, were affected with otitis media, respectively) than in *Eda^Ta^* mice (in which 9 of 24 ears were affected with otitis media). Nonetheless, there was a statistical association between otitis media and otitis externa in individual P21 *Eda^Ta^* ears (Table S1). However, middle ear inflammation was characterised by mild serous effusion with scant leukocytes, whereas inflammation in the ear canal was severe and suppurative, with intralesional cocci; there was no evidence of tympanic membrane perforation or cocci in the middle ear cavity.

### Otitis externa and changes in tympanic membrane and ear canal

The tympanic membrane comprises a rostral (anterior) region, the pars tensa and a posterior region, the pars flaccida, which has a thicker lamina propria. The malleolar fold forms the boundary between the pars tensa and pars flaccida, and is marked by the attachment manubrium of the malleus. The tympanic membrane has an outer squamous keratinising epithelium and a poorly characterised inner mucosal non-keratinising epithelium, which is continuous with the mucosa overlying the bulla bone (Mozaffari et al., 2020). Hereafter, we use the nomenclature relating to tympanic membrane anatomy and histology from Mozaffari et al. (2020).

In the healthy ear, the skin stratified squamous epithelium and the tympanic membrane outer epithelium (pars flaccida and pars tensa) were keratin 5 (K5, also known as KRT5)^+^ (Fig. S3A-C), whereas the simple one-layered inner mucosal epithelium had only scattered K5^+^ cells (Fig. S3B,C). K5 staining occurred in basal cells, suprabasal cells and external root sheath follicular epithelium (Fig. S4B,C).

K7^+^, K8^+^ and K18^+^ (also known as KRT7, KRT8 and KRT18, respectively) cells were present in epithelium of the inner mucosal layer of the pars flaccida and pars tensa, but absent from the outer epithelial layer; Zymbal's gland stained with K5 and K18 (Fig. S3D-L). *In situ* hybridization (ISH) for RNA transcripts encoding the upper respiratory tract innate immunity protein BPIFA1 (Musa et al., 2012) showed that signals were restricted to epithelium of the inner mucosal layer of the pars flaccida and that covering the manubrium of the malleus surface, but not the pars tensa, and the Zymbal's gland was negative (Fig. S3M-O).

P21 *Eda^Ta^* ear canals with tympanic membrane crusts had minor hyperkeratosis in ear canal epithelium (Fig. S4E-G) but this was not noticeable in the tympanic membrane outer epithelium (Fig. S4H). K5-staining was present in basal and suprabasal cells but weak in superficial anucleate squames (Fig. S4F,G). KI67 (also known as MKI67) stained basal epithelial cells in the ear canal and Zymbal's gland, tympanic membrane and inflammatory cells in the bulla cavity (Fig. S4I,J)

In P21 *Eda^Ta^* ear canals with otitis externa, the skin epithelium (Fig. S4K-M) and the outer epithelial layer of the tympanic membrane was thickened and hyperkeratotic (Fig. 2E; Fig. S4N) compared with the normal tympanic membrane (Fig. 2F; Fig. S4D), and K5 staining showed that this epithelium has a porous appearance due to transmural infiltration with polymorphonuclear neutrophils, which stain with the proliferation marker KI67 (Fig. S4L-O). There was no evidence of full-thickness epithelial ulceration or tympanic membrane perforation in any of the P21 mice examined (Fig. 2A-C). Otitis externa in P21 *Eda^Ta^* mice was also associated with inflammation of ear canal dermis (Fig. 2D). Zymbal's gland sebocytes and duct epithelium was K5^+^, and the basal epithelium was KI67^+^ in P21 *Eda^Ta^* mice (Fig. S4P,Q). Zymbal's gland basal cells and mature sebocytes were K5^+^ in P22 *Mecom^Jbo/+^* mice (Fig. S4R,S). The mouse tympanic membrane attains its mature size at P18 (Huangfu and Saunders, 1983), and its dorsal plane diameter was not significantly different in P21 *Eda^Ta^* and P21 FVB mice [median 1.88 mm, 95% c.i. 1.80-2.02 mm (*n*=14), and median 1.88 mm, 95% c.i. 1.81-1.92 mm (*n*=26), respectively; *P*=0.3876, Mann–Whitney test].

### Enrofloxacin treatment of *Eda^Ta^* mice reduces the prevalence of otitis externa and otitis media at P21

Our *Eda^Ta^* mouse colony has a high prevalence of bacterial rhinitis associated with *Staphylococcus aureus* (Azar et al., 2016), and we expected this bacterium would naturally colonise the skin and nasal passages of newborn pups. Enrofloxacin is a broad-spectrum antibiotic used to treat skin and soft-tissue infections in dogs and reaches high concentrations in ear tissues (Cole et al., 2009). We explored the effect of enrofloxacin treatment administered via drinking water (Macy et al., 2000) on ear disease in P21 *Eda^Ta^* mice.

We found *S. aureus* in pure culture in 11 of 12 nasal washes of untreated P21 *Eda^Ta^* mice; one mouse with otitis externa had pure culture of *Aerococcus viridans*. *S. aureus* was found in 14 of 14 nasal washes in enrofloxacin-treated P21 *Eda^Ta^* mice, and the titres were comparable to the untreated group (*P*=0.657, Mann–Whitney test). However, in the enrofloxacin treated group, there was a higher prevalence of mixed cultures with *Enterococcus faecalis* as a co-isolate (5 of 14) compared to the untreated group (0/12) (*P*=0.0425, Fisher's exact test). Furthermore within the enrofloxacin-treated group, *S. aureus* titres were lower when *E. faecalis* was a co-isolate [median 9.0×10^4^ colony-forming units (CFUs), 95% c.i. 8.0×10^3^-4.8×10^5^, *n*=5] than when *S. aureus* was found in pure culture (median 1.0×10^6^ CFUs, 95% c.i. 1.3×10^5^-5.4×10^6^, *n*=9; *P*=0.029, Mann–Whitney test) (Fig. S5K).

Neutrophils were the predominant leukocyte in nasal washes followed by lymphocytes and macrophages (Fig. S5A-C,F,G,J). The number of neutrophils was comparable in enrofloxacin-treated and untreated mice but lymphocytes and macrophages were significantly lower in the treated group (Fig. S5J); squamous cells, basal cells and ciliated cells (Fig. S5E,H,I) were comparable in both groups. Cocci were present in mucus and attached to squamous cells (Fig. S5D,E), and fibres and plant foreign body material were abundant in most samples.

The prevalence of otitis externa and otitis media were significantly reduced in the enrofloxacin-treated group (Table S1, Fig. S2B). There was no evidence enrofloxacin treatment affected Zymbal's gland size or ear canal pilosebaceous unit density in P21 *Eda^Ta^* mice (Fig. 3J,K,R).

### Zymbal's gland hypoplasia and ear canal hypotrichosis in *Edar-*deficient mice

The size of the Zymbal's gland in P82 *Edar-*deficient mice (*Edar^OVE1B/OVE1B^*) was not significantly different than in P82 *Eda^Ta^* mice, and both were significantly smaller than in P82 wild-type mice (Fig. S6). The skin of the osseous ear canal was sparsely haired in *Edar^OVE1B/OVE1B^* and *Eda^Ta^* mice compared with wild-type mice (Fig. S6).

### Zymbal's gland hypoplasia in *Edaradd^swh/swh^* rats

**Fig. 4.**
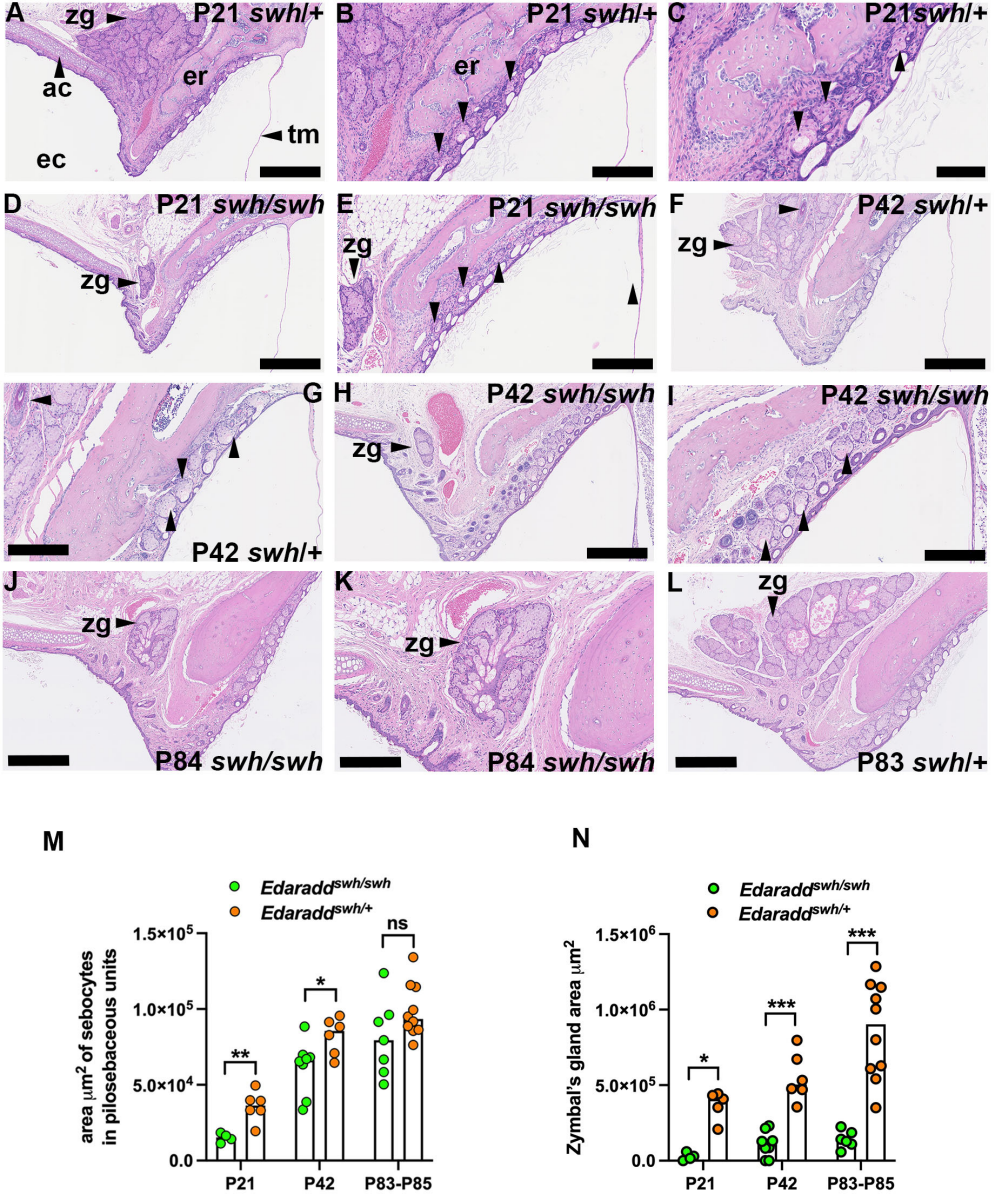
**Pilosebaceous unit sebocytes and Zymbal's gland hypolasia in *Edaradd^swh/swh^* rats.** Dorsal plane sections of ear canal skin in between the tympanic membrane and the annular cartilage in *Edaradd^swh/swh^* and *Edaradd^swh/^*^+^ rats. Panel orientation: left is lateral, right medial, top rostral and bottom caudal. Panel groups A, B and C, D and E, F and G, H and I, and J and K are images of the same section at different magnifications. Pilosebaceous units are indicated by unlabelled arrowheads. (A-I) The ear canals of *Edaradd^swh/swh^* and *Edaradd^swh/^*^+^ rats have a similar density of pilosebaceous units. In F and G, a P42 *Edaradd^swh/swh^* rat has a large hair follicle adjacent to the Zymbal's gland (unlabelled horizontal arrowheads). (J,K) Zymbal's gland hypoplasia in a P84 *Edaradd^swh/swh^* rat. (L) P83 *Edaradd^swh/^*^+^ rat with a normal Zymbal's gland. (M,N) Morphometric analysis. (M) Sebocytes in ear canal pilosebaceous units are reduced in P21 and P42 *Edaradd^swh/swh^* rats compared with *Edaradd^swh/+^* heterozygous littermate controls but comparable at P83-P85. (N) Zymbal's gland size is reduced in P21, P42 and P83-P85 *Edaradd^swh/swh^* rats compared with *Edaradd^swh/+^* heterozygous littermate controls. Data are represented as points and the histogram bar as the median. *P<0.05; **P<0.01; ***P<0.001; ns, not significant (two-tailed Mann–Whitney test). ac, annular cartilage; ec, ear canal; er, ectotympanic ring; tm, tympanic membrane; zg, Zymbal's gland. Scale bars: 500 µm (A,D,F,H,J,L); 250 µm (B,E,G,I,K); 100 µm (C).

Zymbal's glands in *Edaradd^swh/swh^* rats were significantly smaller than those in *Edaradd^swh/^*^+^ rats at P21, P42 and P83-P85 (Fig. 4A,D,F,H,J-L,N). There was no overt ear canal hypotrichosis in *Edaradd^swh/swh^* rats compared to *Edaradd^swh/^*^+^ rats (Fig. 4B,C,E,G,I). Pilosebaceous unit sebocytes were reduced at P21 and P42 but were comparable to littermate *Edaradd^swh/^*^+^ rats at P83-P85 (Fig. 4M). The tympanic membrane diameters were comparable in P21 *Edaradd^swh/swh^* (median 2.90 mm, 95% c.i. 2.53-3.19 mm, *n*=3) and littermate *Edaradd^swh/^*^+^ rats (2.93 mm, 95% c.i. 2.57-3.14 mm, *n*=5; *P*=0.7857, Mann–Whitney test). Otitis externa was not observed in *Edaradd^swh/swh^* (P21, *n*=6 ears; P30-P85, *n*=26 ears), *Edaradd^swh/^*^+^ (P21, *n*=8 ears; P30-P85, *n*=20 ears) or *Edaradd*^+/+^ rats (P83-P85, *n*=4 ears).

### *Edar* expression in Zymbal's glands

**Fig. 5.**
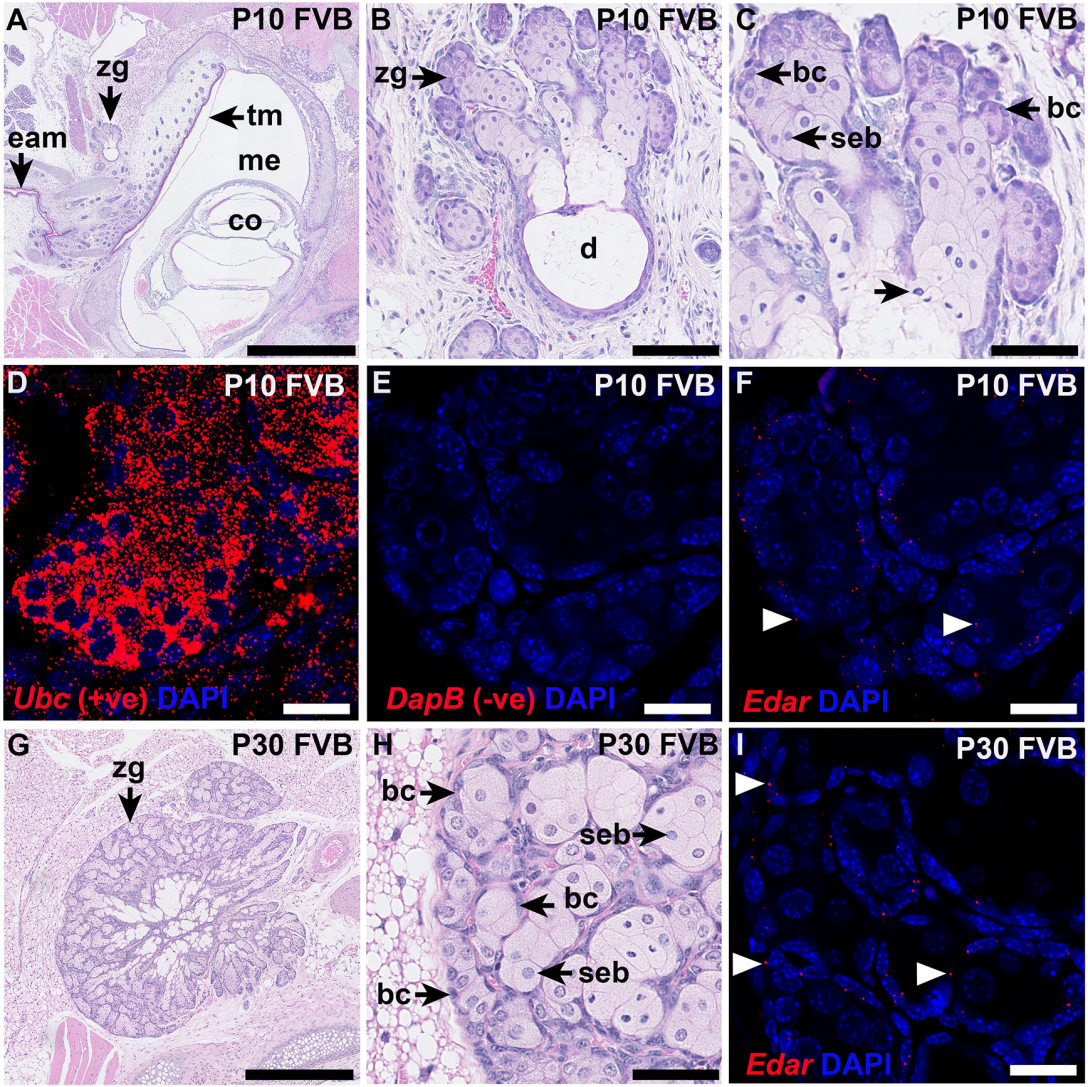
***Edar* expression in FVB Zymbal's gland.** (A-C) Dorsal plane images of P10 FVB Zymbal's gland are different magnifications of the same section (H&E stain). (A,B) Sebaceous tissue lobules in the immature gland connect with a widely patent duct. (C) Basal cells give rise to mature sebocytes, which secrete in a holocrine manner (unlabelled arrowhead) (H&E stain). (D-F) Nearby serial sections of Zymbal's gland are fluorescent ISH preparations with DAPI nuclear counter stain. (D) The positive control probe (*Ubc)* shows intense ISH signals in all gland cell types and adjoining connective tissue. (E) ISH signals are absent with the negative control probe (*DapB*). (F) *Edar* signals are punctate spots chiefly associated with Zymbal's gland basal cells (arrowheads) rather than centrilobular sebocytes. (G,H) H&E-stained parasagittal sections of P30 Zymbal's gland. Panel I is a higher power magnification of the same section showing gland basal cells and mature sebocytes. (I) Nearby serial section of Zymbal's gland showing that *Edar* ISH signals (arrowheads) are chiefly associated with Zymbal's gland basal cells. The ISH images were all acquired using the same microscope settings. bc, basal cell; co, cochlea; d, duct; eam, external auditory meatus; tm, tympanic membrane; seb, sebocyte; zg, Zymbal's gland. Scale bars: 1 mm (A); 500 µm (G); 100 µm (B); 50 µm (C,H); 20 µm (D-F,I).

ISH of adult wild-type mouse skin showed *Edar* expression in basal cells and, to a lesser extent, sebocytes of follicle-associated sebaceous glands (Kowalczyk-Quintas et al., 2015). *Edaradd* is expressed in the follicle-associated sebaceous glands of the P13 *Edaradd^swh/^*^+^ rat and in the Zymbal's gland of the P10 *Edaradd*^+/+^ rat (del-Pozo et al., 2019a). As the postnatal growth of the mouse Zymbal's gland appears to be dependent on EDAR signalling, we investigated which cell types expressed *Edar.* In P10 (Fig. 5A-F) and P30 (Fig. 5G-I) FVB mice, we found the greatest density of punctate *Edar* ISH signals in basal cells at the periphery of the Zymbal's gland lobules (Fig. 5F,I), whereas the positive control probe, detecting ubiquitin C (*Ubc*), showed signals were abundant in all gland cell types (Fig. 5D). No signal was detected by the negative control probe, which recognises the bacterial *DapB* gene (Fig. 5E).

*Edar* ISH signals were also detected in the P21 *Eda^Ta^* Zymbal's gland; however, the gland is relatively small and has less pronounced lobulation than the wild-type FVB gland (Fig. S7). Both the *Edar* signal and the positive control probe in P21 *Eda^Ta^* (Fig. S7) were relatively lower than in P10 FVB (Fig. 5), but the interpretation of relative expression levels in these separate experiments is problematic as the samples were produced in different labs, and would likely have had differences in gland anatomy and its constituent cell populations.

## DISCUSSION

We report that *Eda^Ta^* and *Edar^OVE1B/OVE1B^* mice, and *Edaradd^swh/swh^* rats, have growth retardation of the Zymbal's gland, resulting in gland hypoplasia, emphasizing the general importance of the EDA-EDAR signalling pathway in rodent Zymbal's gland development. The development of Zymbal's gland has not been investigated in detail but in *Eda^Ta^* and NF-kB^−/−^ mice, the Meibomian and preputial glands do not develop, and they are reduced in Traf6^−/−^ mice, indicating the involvement of Troy (also known as TNFRSF19) signalling, as well as EDA-EDAR signalling (Dhouailly and Oftedal, 2016). Zymbal's gland development is considered to be independent of hair follicle development (Dhouailly and Oftedal, 2016), and in keeping with this, we found small sebaceous gland rudiments in P21 *Eda^Ta^* located in the normal anatomical site of Zymbal's gland. These are larger than normal ear canal pilosebaceous unit sebaceous glands, lack obvious hair follicles and develop into histologically normal Zymbal's glands by P79-P90. We observed only a single instance of a vibrissa-sized hair follicle located in Zymbal's gland tissue of an *Edaradd^swh/swh^* rat among the hundreds of histological sections screened in this study.

The embryonic Zymbal's gland primordium is present at E15 (Grüneberg, 1971), and we found that prenatal (at E10.5 and E17.5) and prenatal and postnatal treatment (at E10.5, E17.5, P1, P7, and P14) of *Eda^Ta^* mice with EDAR signalling agonist antibody (del-Pozo et al., 2019a) promotes Zymbal's gland growth by P21. We found *Edar* gene expression in the Zymbal's gland of P10 and P30 FVB mice, as well as in P21 *Eda^Ta^* mice, suggesting the adult gland has the potential to respond to EDAR signalling. These results are consistent with the known importance of EDAR signalling in the development and function of sebaceous glands, and Meibomian glands in mice, rats, dogs and humans (Kuramoto et al., 2005, 2011; Kaercher et al., 2014; Kowalczyk-Quintas et al., 2015; Waluk et al., 2016; Schneider et al., 2018; Margolis et al., 2019).

The Zymbal's glands continue to grow in untreated *Eda^Ta^* mice, and by P79-P90 the glands attain ∼50% of normal size, and prenatal agonist anti-EDAR antibody treatment did not result in additional growth in older mice. Sustained treatment of *Eda^Ta^* and wild-type mice with agonist anti-EDAR antibody heightens sebum production by hair follicle sebaceous glands (Kowalczyk-Quintas et al., 2015), and we infer that continuous treatment would have the same effect on the adult Zymbal's gland. A single treatment with agonist antibody at P21-P26 produces a long-lived but reversible effect on *Eda^Ta^* hair follicle sebaceous gland size; the effect was present at 12 weeks but not after 24 weeks (Kowalczyk-Quintas et al., 2015). The apparent lack of effect on P79-90 *Eda^Ta^* Zymbal's gland size by *in utero* treatment may represent a similar time-limited response to agonist antibody treatment. Zymbal's gland growth was also reduced (to ∼46% of normal size) in P82 *Edar*-deficient mice compared to P82 wild-type mice.

In *Eda^Ta^* and *Edar^OVE1B/^*^OVE1B^ mice, but not *Edaradd^swh/swh^* rats, there is marked ear canal hypotrichosis. This difference between the density of ear canal hair follicles in the rat and mouse model mirror those elsewhere in the body. *Edaradd^swh/swh^* rats have sparse hair coats and hypoplasia of pilosebaceous units in skin of the head, dorsum and ventrum, but unlike *Eda^Ta^* mice, *Edaradd^swh/swh^* rats have a haired tail and lack a bald patch behind the ear (Kuramoto et al., 2005, 2011). P10 *Eda^Ta^* mice have few ear canal hair follicles, and these have small sebaceous glands (Grüneberg, 1971). Additionally, hair follicles in the adult pinna have small sebaceous glands (Kowalczyk-Quintas et al., 2015). The scarcity of pilosebaceous units in the ear canal may exacerbate the regional deficit of sebum caused by Zymbal's gland hypoplasia. Although EDAR signalling deficiency in *Edaradd^swh/swh^* rats results in marked Zymbal's gland hypoplasia, ear canal pilosebaceous units were less affected.

Six of 12 P21 *Eda^Ta^* mice had unilateral otitis externa, but this was absent in P21 FVB mice with full-sized Zymbal's glands, P21 *Eda^Ta^* mice with rescued Zymbal's glands and in P22 *Mecom^Jbo/+^* and P21 *Fbxo11^Jf/+^* mice, which have unremarkable Zymbal's glands. Additionally, otitis externa was absent in P79-P90 *Eda^Ta^* mice (untreated, and those administered agonist anti-EDAR or isotype antibodies).

Gram-positive cocci were found in all otitis externa lesions, and *S. aureus* is a candidate pathogen (this study and Azar et al., 2016). There are potential similarities here with the nasal carriage of *S. aureus* being a risk factor for the development of human skin infection (Toshkova et al., 2001). Treatment of mice with enrofloxacin via 0.25 mg/ml drinking water gives a peak plasma level of 140 ng/ml plasma (Marx et al., 2014), and this dosing regimen in *Eda^Ta^* dams and their pups reduced the prevalence of otitis externa and otitis media at P21. The required minimum inhibitory concentration (MIC)_90_ for *S. aureus* is 120-250 ng/ml and for *Enterococcus* spp is 1000-2000 ng/ml (Marx et al., 2014). The concentrations of enrofloxacin and its metabolite ciprofloxacin in skin, inflamed ear canal tissue and middle ear in dogs with end-stage otitis externa and intercurrent otitis media are significantly higher than those in plasma (4-11-fold and 2-6-fold for enrofloxacin and ciprofloxacin, respectively) (Cole et al., 2009). In addition, enrofloxacin and ciprofloxacin are lipophilic (Blokhina et al., 2016), and are likely to concentrate in sebum lipid, thereby providing antimicrobial prophylaxis that compensates for smaller Zymbal's glands. Although enrofloxacin treatment did not reduce overall nasal bacterial load in P21 *Eda^Ta^* mice, there were subtle changes, such as an increased prevalence of resistant *E. faecalis* as a co-isolate with *S. aureus* and reduced lymphocyte and macrophage populations. The change in leukocyte differentials may represent a delay in progression toward chronic inflammation and an adaptive immune response.

The microbial status of *Eda^Ta^* mice also plays an important role in eye disease presentation and progression. Conventionally housed (low health status) and specific pathogen-free (SPF) (high health status) *Eda^Ta^* mice have Meibomian gland deficits that result in corneal defects caused by desiccation and mechanical injury. However, inflammation of the eyelids (blepharitis) and conjunctiva (conjunctivitis) were only observed in conventionally housed mice (Cui et al., 2005).

The pathogenesis of tympanic membrane crusts in *Eda^Ta^* mice is unclear. There are some similarities with tympanic membrane inflammatory casts that are described in humans. These consist of hardened fibrin with low inflammatory cell content, and are hypothesised to be derived from serous exudate from acute otitis media with tympanic membrane perforation or otitis externa (Byun et al., 2013). The similarity in composition between *Eda^Ta^* crusts and otitis externa exudates raises the possibility that crusts represent an early or resolving phase of ear canal infection and inflammation.

Pre-weaning (2-week-old) mice are more susceptible to *S. aureus* sepsis due to reduced neutrophil chemotaxis and macrophage phagosome maturation (Zhang et al., 2013). The absence of otitis externa in wild-type mice and in *Eda^Ta^* mice with rescued Zymbal's glands indicates that myeloid cell immaturity alone does not initiate otitis externa. Nevertheless, functional immaturity could be a contributory factor to otitis externa susceptibility, and conversely, mature myeloid cell function may aid its resolution. There are no identified humoral or cellular immune deficits in HED that might contribute to otitis externa susceptibility. Myeloid and lymphocyte cell classes in blood, spleen, bone marrow and peritoneum are broadly similar in adult EDAR-deficient *downless* mice (*Edar^dlJ/dlJ^*) and unaffected heterozygous littermates (*Edar^dlJ/^*^+^), and the phagocytic activity of peritoneal macrophages is comparable in *Eda^Ta^*, *Edar^dlJ/dlJ^* and *Edar^dlJ/^*^+^ mice (Azar et al., 2016). Furthermore, dogs with XLHED have frequent respiratory tract infections but this is attributable to reduced mucociliary clearance and the absence of bronchial glands rather than any identifiable immune deficiency (Casal et al., 2005). *Eda^Ta^* mice did not have congenital stenosis of the ear canal at the level of tympanic membrane attachment to the tympanic ring. However, there is minor canal wall thickening during acute inflammation.

We interpret that the pattern of ear canal infection and inflammation in *Eda^Ta^* mice is primarily the result of changes in Zymbal's gland size and function, which occurs as follows: (1) the window of susceptibility to bacterial infection and initiation of otitis externa occurs in the interval between the re-opening of the ear canal at P7 and P21, during which Zymbal's gland growth in the absence of EDA is retarded; (2) endemic skin/nasal commensal bacteria, such as *S. aureus*, act as opportunistic pathogens; (3) Zymbal's gland hypoplasia and ear canal hypotrichosis at ≤P21 results in sebum deficiency and thereby reduced innate immune protection (see discussion below); (4) agonist anti-EDAR treatment promotes Zymbal's gland growth and sebum production by P21 to reduce susceptibility; (5) EDAR signalling-independent growth of the Zymbal's gland between P21 and P79 restores sebum protection; and (6) the absence of otitis externa in older mice indicates that acute disease is self-limiting and that tissue injury is repaired without identifiable sequelae, such as ear canal stenosis.

The numbers of *Edaradd^swh/swh^* rats in this study were too low to fully assess the prevalence of otitis externa but there are reasons to suspect that rats may be less susceptible. The sebum secreted by ear canal pilosebaceous units in *Edaradd^swh/swh^* rats may provide sufficient protection and/or infection by opportunistic pathogens is less common. Nasal bacteria may be a significant source in *Eda^Ta^* mice, and it is noteworthy that P21-P85 *Edaradd^swh/swh^* rats do not have rhinitis (del-Pozo et al., 2019a). A microbial challenge experiment might be a useful approach to explore infection of the ear canal in *Edaradd^swh/swh^* rats. Otitis externa was not observed in the small number of *Edar^OVE1B/OVE1B^* mice examined in this study. Reduced Zymbal's gland size and ear canal hypotrichosis may well predispose to ear canal infection in *Edar^OVE1B/OVE1B^* mice but spontaneous disease will also depend on the occurrence of opportunistic bacterial pathogens in colony mice.

The response of the tympanic membrane to otitis externa is not well documented but its barrier function and resilience would presumably depend on epithelial integrity and regenerative capacity. We found that the outer keratinising epithelial layer is K5^+^ but the inner mucosal layer epithelium has only scattered K5^+^ cells, which may represent a putative stem cell population. K5 is abundant in basal cells of human stratified squamous epithelium and downregulated in suprabasal cell layers (Moll et al., 2008). We observed suprabasal K5 staining in healthy, mildly hyperkeratotic and inflamed hyperkeratotic ear canal epithelium. The stratified epithelium of ear canal is thicker than that of the tympanic membrane, and number differentiating cell layers, as well as increased cell proliferation, appear to contribute to variation in suprabasal cell expression of K5 protein. The primary keratin pair K8 and K18, and the secondary keratin K7, are expressed in simple (one-layered) epithelium (Moll et al., 2008), and we found them expressed in the inner mucosal epithelial layer of the tympanic membrane. The pattern of keratin and BPIFA1 expression in the inner epithelial layer is similar to that of the non-tympanic middle ear epithelium (del-Pozo et al., 2019b); however, BPIFA1 is restricted to the epithelium of the pars flaccida and the malleus. Bacterial infection of the ear canal stimulates recruitment of neutrophils and these are observed transiting through the hyperkeratotic outer epithelium of the tympanic membrane and the ear canal.

In P21 *Eda^Ta^* ears there is a statistical association between otitis media and otitis externa but tympanic membrane perforation was not observed. Ear canal infection is characterised by severe inflammation and intralesional cocci, so it is unlikely to be secondary to mild middle ear inflammation where cocci are absent. Even when otitis media is severe in >P79 *Eda^Ta^* mice there is no evidence of otitis externa. We conclude that the two conditions develop independently in P21 *Eda^Ta^* mice (otitis media via an auditory tube gating defect, del-Pozo et al., 2019b), and that otitis media does not predispose to otitis externa in *Mecom^Jbo/+^* and *Fbxo11^Jf/+^* mice.

In contrast to *Eda^Ta^* mice, ear canal pathology in human HED is characterised by cerumen impaction and stenosis (Siegel and Potsic, 1990; Daniel et al., 2002; Shin and Hartnick, 2004; Mehta et al., 2007; Yildirim et al., 2012; Callea et al., 2013). It is possible that cerumen impaction in human HED is a manifestation of impaired sebaceous and ceruminous gland secretion, and failure of keratinocyte expulsion from the ear canal.

Human cerumen comprises desquamated keratinocytes and ∼50% of the dry weight is a lipid fraction, comprising long-chain fatty acids, triacylglycerols, cholesterol and cholesterol esters, wax esters, ceramides and squalene. The squalene and wax esters appear to be sebaceous gland lipids rather than squamous cell products (Bortz et al., 1990; Guest et al., 2004). Although impacted cerumen is a rich medium for microbial growth (Guest et al., 2004), the modification of sebum triglycerides by bacteria produces free fatty acids, and the action of epithelial ceramidases on ceramides produces sphingosines; both free fatty acids and sphingosines have antimicrobial activity (Wertz, 2018). In a study by Lum et al. (2009) >87% of human cerumen samples had bactericidal activity against *S. aureus* and *Pseudomonas aeruginosa*, and fungicidal activity against *Candida albicans* but a minority of samples promoted growth. Proteomic analysis of cerumen has identified antimicrobial constituents such as zinc-alpha-2-glycoprotein, cathepsin D, apo-lipoprotein D, serpins, calpain, mucins and lysozyme C (Feig et al., 2013). The tympanic membrane and pilosebaceous units in the human ear canal produce beta defensins (hBD1 and hBD2) (Bøe et al., 1999; Yoon et al., 2008), whereas ceruminous (apocrine) glands produce hBD1, hBD2, cathelicidin, lysozyme, lactoferrin, MUC1 and the secretory component of IgA (Stoeckelhuber et al., 2006).

Current animal models of AOE depend on disrupting the ear canal epithelial barrier. A Sprague Dawley rat model of otitis externa has had mechanical abrasion of the cartilaginous ear canal induced, and the resultant inflammation, dermal thickening and hyperkeratosis can be moderated by topical application of steroid or polymyxin B (Emgård and Hellström, 1997, 2001). This model was also infected with *P. aeruginosa* or *C. albicans* to demonstrate the efficacy of topical steroids without antibiotics (Emgård et al., 2005) and thymoquinone (Demirel et al., 2018). A guinea pig model of infectious otitis externa has been established by perturbing the ear canal environment and its commensal bacteria, which facilitates experimental *P. aeruginosa* infection (Wright and Dineen, 1972). Unlike other AOE models, gerbils were successfully infected with *Klebsiella pneumoniae* without ear canal pretreatment (Zhai et al., 2014). Topical application of tetradecanoylphorbol acetate induces acute skin inflammation, and has been used in the mouse ear canal to evaluate the efficacy of ciprofloxacin and hydrocortisone ear drops in reducing inflammation (Wright et al., 2000).

The first step toward developing an *Eda^Ta^* model of otitis externa will be to establish an inoculation protocol and to gain a better understanding of the time course and its spontaneous resolution. If ear canal infection rates can be raised with microbial challenge, the numbers of mice used will be minimised. It may be necessary to establish an *Eda^Ta^* mouse colony with low nasal commensal bacteria through the use of prophylactic antibiotics to avoid interference with experimental microbial inoculations. The advantages of an *Eda^Ta^* mouse challenge model are that inoculation requires no pretreatment of the ear canal and is therefore minimally invasive, husbandry costs are minimised by having a short bioassay time and otitis externa appears to be a self-limiting disease. However, there are noteworthy species differences, in particular the apparent lack of apocrine ceruminous glands in rodents, which may affect cerumen composition and consistency. The issue of whether otitis externa causes otalgia needs to be addressed, but there was no obvious self-inflicted pinna injury, and ear canal inflammation was localised.

In conclusion, we report that mouse and rat models of HED have Zymbal's gland hypoplasia, and in *Eda^Ta^* mice there is also ear canal hypotrichosis. Sebum deficiency coupled with endemic nasal carriage of *S. aureus* in our SPF colony predisposes to opportunistic infection of the ear canal and AOE in P21 *Eda^Ta^* mice. To our knowledge, this is the first report of naturally occurring otitis externa in inbred or genetic strains of laboratory mice. The *Eda^Ta^* mouse is a model of AOE that will be useful for investigating the cellular and molecular pathology of ear canal infection.

## MATERIALS AND METHODS

### Animals and *in vivo* procedures

The animal experiments were reviewed and agreed by the Roslin Institute Animal Welfare and Ethical Review Body, and were performed under the authority of an appropriate UK Home Office Licence. Tabby mice (*Eda^Ta/Ta^* females and *Eda^Ta/^*Y hemizygous males; collectively termed *Eda^Ta^*) were maintained as a homozygous line. FVB mice are the background inbred genetic line for the *Eda^Ta^* strain and FVB/NCrl (Charles River) mice were bred to provide control tissues. The sparse and wavy hair (swh) *Edaradd* rat strain (Kuramoto et al., 2005, 2011) WTC-swh/Kyo [National BioResource Project (NBRP) Rat No. 0287] was supplied by the NBRP - Rat, Kyoto University, Japan. The *Edaradd* rat colony was maintained by mating heterozygous *Edaradd^swh^*^/+^ rats, or mating male *Edaradd^swh^*^/+^ with *Edaradd^swh/swh^* females. The heterozygote *Edaradd^swh/+^* rat has a wild-type appearance but homozygous *Edaradd^swh/swh^* animals have typical HED dental and cutaneous phenotypes.

Heterozygous *Fbxo11^Jf/+^* mice [Mouse Genome Informatics (MGI), 1862017; European Mouse Mutant Archive (EMMA), EM:00375] and their *Fbxo11*^+/+^ wild-type littermates were generated by intercrossing F1 *Fbxo11^Jf^*^/+^ C57BL/6J C3H/HeH males with C57BL/6J (Charles River) females. Heterozygous *Mecom^Jbo/+^* mice (MGI, 2158381; EMMA, EM:00091) and their wild-type littermate controls, *Mecom^+/+^*, are congenic on a C3H/HeH genetic background. These strains were obtained from the Mary Lyon Centre (Medical Research Council, Harwell). Mouse and rat husbandry, genotyping, health surveillance and SPF status are reported elsewhere (Azar et al., 2016; del-Pozo et al., 2019a,b). Male and female mice and rats were used in all analyses.

White-bellied agouti B6CBAa *A^w-J^/A-Eda^Ta^/*J Tabby mice (000314; Jackson Laboratory) were bred as *Eda^Ta^*/*Eda^Ta^* and *Eda^Ta^*^/^Y mutants or as −/− and −/Y wild-type controls. EDAR-deficient OVE1B mice were bred as *dl^Ove1B^/dl^Ove1B^* (described previously by Headon and Overbeek, 1999). Four to five animals per cage were housed in an SPF facility at 21°C and 50±10% humidity, with a 14 h-10 h light/night cycle. Mice were provided *ad libitum* with water at pH 2.8 and Global Rodent XP18 food (Kliba Nafag). Cages were enriched with tunnel kraft, virgin cellulose home, sizzle pad 8G and aspen and beech brick (Serlab). These strains were bred at the University of Lausanne. All mice were handled according to the Swiss Federal Veterinary Office guidelines, under the authorization of the Office Vétérinaire Cantonal du Canton de Vaud (authorization 1370.8 to P.S.).

*Eda^Ta^* mice were administered agonist anti-EDAR antibody (mAbEDAR1) (Kowalczyk et al., 2011; Schuepbach-Mallepell et al., 2021) at 2 mg/kg either prenatally (E10.5 and E17.5) (*n*=2) or prenatally and postnatally (E10.5, E17.5, P1, P7 and P14; *n*=6), and phenotyped at P21. In addition, *Eda^Ta^* mice treated prenatally (E10.5 and E17.5) with anti-EDAR antibody (*n*=9) or with isotype control antibody (Aprily2) (*n*=10) were phenotyped at P85 or P82-P84, respectively (see details of administration routes in del-Pozo et al., 2019a).

Two *Eda^Ta^* dams were administered enrofloxacin via drinking water (25 mg/ml injectable form of Bayrtril, Bayer, Shawnee Mission, KS, USA) throughout pregnancy and lactation (Macy et al., 2000; Towne et al., 2014), and based on water intake, this is equivalent to a dosage of 40 mg/kg. Enrofloxacin, like other drugs, is secreted into milk in humans, mice and bovines via the ABCG2 efflux transporter (Jonker et al., 2005), and enrofloxacin and its metabolite ciprofloxacin are secreted in bovine milk (Idowu et al., 2010). Taken together, mouse pups are likely to acquire antibiotic through milk feeding, as well as by drinking water for themselves once they begin to eat solid food from P12 onwards.

Two litters born to enrofloxacin-dosed *Eda^Ta^* dams (*n*=5 and *n*=9 pups) and two litters born to dams provided with normal drinking water (*n*=5 and *n*=7 pups) were euthanised at P21. Mice were sampled mortem by nasal wash microbiology and cytology as described previously (Azar et al., 2016), and the heads were prepared for histology.

### Histology and morphometric analysis

Animals were euthanised with a rising concentration of CO_2_ then decapitated, and the skin, outer pinnae and brain removed. This dissection leaves the ear canal and Zymbal's gland intact. The fixation of tissues in neutral buffered formalin (NBF) and preparation of the decalcified and wax-embedded heads used in this study, along with details of sectioning in the dorsal plane, staining procedures for Haematoxylin and Eosin (H&E), immunohistochemistry for KI67, and ISH for cytokeratins K5, K7, K8 and BPIFA1, have been reported previously (del-Pozo et al., 2019a,b).

K18 immunohistochemistry was performed with rabbit anti-cytokeratin 18 antibody (monoclonal EPR17347, Abcam, ab181597) diluted 1 in 1200 and applied for 30 min at room temperature. Antigen retrieval was carried out using Tris-EDTA buffer (pH 9.0) at 110°C for 5 min, and primary antibody binding was detected using Rabbit Envision (Dako) for 40 mins and DAB chromogen. Additional special stains included Masson's trichome, Gram Twort, PAS and Grocott's methenamine silver stain. Histology and immunohistochemistry were performed at the Easter Bush Pathology laboratories, which are UK National External Quality Assessment Service accredited.

Bright-field images were acquired using an Olympus BX41 microscope equipped with a DP72 camera and Cell D software. Slide scans were made using a Hamamatsu NanoZoomer. Morphometric analysis for object length and area was performed using NanoZoomer software and QuPath software (Bankhead et al., 2017), respectively.

The Zymbal's gland is located on the anterior (rostral) ear canal between the ectotympanic ring bone and the annular cartilage. We scored the presence or absence of the Zymbal's gland and measured its total area (sebaceous glandular lobules, connective tissue, ducts and their lumens). P21 *Eda^Ta^* mice have small sebaceous gland lobules in the normal location for Zymbal's gland, and we interpret these to be Zymbal's gland rudiments rather than of pilosebaceous origin because they were not associated with hair follicles. Any gland tissue microscopically associated with a hair follicle was excluded from the assessment. We also measured the size of sebaceous glands in P21 FVB ear canal pilosebaceous units to compare with the size of sebaceous gland lobules in P21 *Eda^Ta^* mice.

We measured Zymbal's glands at a standardised dorsal plane in a ∼0.4-cm zone between the lateral opening of the ear canal where the Zymbal's gland has its largest profile, upward to the level of the cochlea round window. In FVB samples (P21 and P81-P84 ears), a single Zymbal's gland profile measurement per ear was adequately representative. In P21 *Eda^Ta^*, the Zymbal's glands are smaller and not always evident in random sections, so we assessed multiple 40-µm step sections (technical replicates) and averaged two to five measurements. For consistency, we also sampled in this way for P79-P90 *Eda^Ta^* ears.

In addition, we measured Zymbal's gland size in dissected P82 ear canals of wild-type (*n*=8) controls, *Eda^Ta^* (*n*=5) and *Edar*-deficient mice (*Edar^OVE1B/OVE1B^*) (*n*=3) from the University of Lausanne colony. Ear canals were serially sectioned in 100-µm steps, and we averaged two to six area measurements.

We also averaged Zymbal's gland area in two to five step sections in P21, P42 and P83-P85 *Edaradd^swh/swh^* and *Edaradd^swh/+^* rats. In P83-P85 *Edaradd^swh/+^* rats the Zymbal's gland can have cystic intralobular ducts and these cystic spaces were omitted from the gland area measurements.

Ear canal pilosebaceous units (the hair follicles and associated sebaceous glands) were counted on the rostral surface of ectotympanic bone between the tympanic membrane and the beginning of the annular cartilage in P21 and P79-P90 *Eda^Ta^*, P21 and P81-84 FVB mice from the Roslin animal colony, and P82 wild-type, P82 *Eda^Ta^* and P82 *Edar^OVE1B/OVE1B^* mice from the University of Lausanne colony. Each data point is the median of 2-6 serial step sections.

In addition, we measured the area of sebocytes (excluding ducts and connective tissue) in the ear canal pilosebaceous units of P21, P42 and P83-P85 *Edaradd^swh/swh^* and *Edaradd^swh/+^* rats using the same landmarks. Skin over the annular cartilage and osseous regions of the ear canal were examined in serial step sections of P21 FVB mice (*n*=6) for the presence or absence of apocrine glands.

The thickness of ear canal soft tissue (epithelium, dermis and periosteum) overlying the rostral surface ectotympanic ring bone was measured in P21 FVB and in P21 *Eda^Ta^* mice. The area of tissue was divided by the length of the underlying bone to calculate its average thickness.

The ear canal was scored for presence or absence of otitis externa or thin crusts overlying the tympanic membrane. The diagnostic criterion for otitis externa was the presence of a substantial amount of inflammatory exudate filling the ear canal base adjacent to the tympanic membrane. The exudate is composed of neutrophils mixed with eosinophilic amorphous debris, exfoliated squamous epithelial cells and Gram-positive cocci. This is accompanied by hyperkeratosis and inflammatory thickening of the tympanic membrane and ear canal dermis. Thin crusts overlying the tympanic membrane have a similar composition but the tympanic membrane is not substantially thickened, and although the ear canal epithelium can be hyperkeratotic, intraepithelial inflammatory cell infiltration and dermal inflammation are absent.

The tympanic membrane dorsal plane diameter was measured between its fibrocartilaginous insertion points. We averaged two to five 40-µm step sections for each tympanic membrane length measurement in P21 *Eda^Ta^* (unaffected by otitis externa), P21 FVB mice and in P21 *Edaradd^swh/swh^* and P21 *Edaradd^swh/+^* rats. The middle ear bullae were scored for the presence or absence of otitis media, characterised by the presence of inflammatory cells in the bulla cavity and thickening of the mucosa.

We examined the heads of P21 (*n*=13) and P81-84 (*n*=10) FVB mice; P21 (*n*=12) and P79-P90 (*n*=13) *Eda^Ta^* mice; P22 *Mecom^Jbo/+^* mice (*n*=7); P21 *Fbxo11^Jf/+^* (*n*=8); and P21 (*n*=3), P30 (*n*=5), P42 (*n*=4), P83-P85 (*n*=4) *Edaradd^swh/swh^* and P21 (*n*=4), P30 (*n*=1), P42 (*n*=4), P83-P85 (*n*=5) *Edaradd^swh/+^* rats.

Not every ear section was suitable for making all measurements and assessments, and exclusion criteria were incomplete sections through accidental overtrimming of decalcified tissue blocks, the plane of section being outside target level, or processing artefacts that obscure critical features. The number of ears (biological replicates) assessed for each feature are given in the results and figure legends. Additional mice were surveyed for the presence or absence of otitis externa: P84 *Mecom^Jbo/+^* (*n*=6), P22 *Mecom*^+*/+*^ (*n*=7); P57-P223 *Fbxo11^Jf/+^* (*n*=22); and *Fbxo11*^+*/+*^ (*n*=5).

### *Edar in situ* hybridization

The skulls of P10, P21 and P30 mice were skinned, and brains were removed and fixed in NBF. The ear canals were dissected within 1-2 h, then returned into fixative for a total time of 24 h then decalcified with 14% EDTA for 8 h (P10) or 12 h (P21 and P30) before processing to wax.

Edar ISH was performed manually using an RNAscope Multiplex Fluorescent V2 assay (ACD). Briefly, paraffin-embedded tissue sections (4 µm) were prepared and processed as per the manufacturer's instructions. Sections were hybridised with a mouse Edar probe (ACD, 423011-C3), and serial sections were incubated with either a species-specific 3-plex positive control (ACD, 320881) or a 3-plex negative control (ACD, 320871). Following hybridisation and subsequent amplification steps, sections were incubated with Opal 620 dye (Akoya Biosciences, FP1495001KT), counterstained with DAPI and mounted in Prolong Gold. Samples were imaged using a Zeiss LSM 880 confocal microscope.

### Statistical analysis and graphical representation

D'Agostino–Pearson normality tests showed the datasets were not normally distributed (or that the group size was too small to test for normality), so we used either Mann–Whitney tests or Kruskal–Wallis tests followed by Dunn's multiple comparison tests. Graphs represent data as either points with the median, or Tukey's method box-and-whisker plots; the box represents the 25% percentile, the median and the 75% percentile, the whiskers represent the minimum and the maximum, and the outliers are represented as points.

Contingency tests comparing disease frequencies were performed using Fisher's exact tests. Ear disease frequency can be expressed by two measures, per animal and per ear. As ear disease was often unilateral, it follows that the frequency of affected animals is higher than the frequency of affected ears. For this reason, disease frequency per animal may achieve statistical significance but the same data may not show a significant difference per ear. The raw data and the contingency test results are presented in Table S1. Graphs and statistics were generated using GraphPad Prism version 8.4.3 (471). Two-tailed tests were used throughout and test values of *P*<0.05 were considered to be statistically significant.

## Supplementary Material

Supplementary information
