# Supplementary material for: The EDA-deficient mouse has Zymbal's gland hypoplasia and acute otitis externa
Source: Dis Model Mech. 2022 Mar 30;15(3):dmm049034. doi: 10.1242/dmm.049034 (PMC8990926; doi:10.1242/dmm.049034)
Supplement: Supplementary information [file dmm-15-049034-s1.pdf]

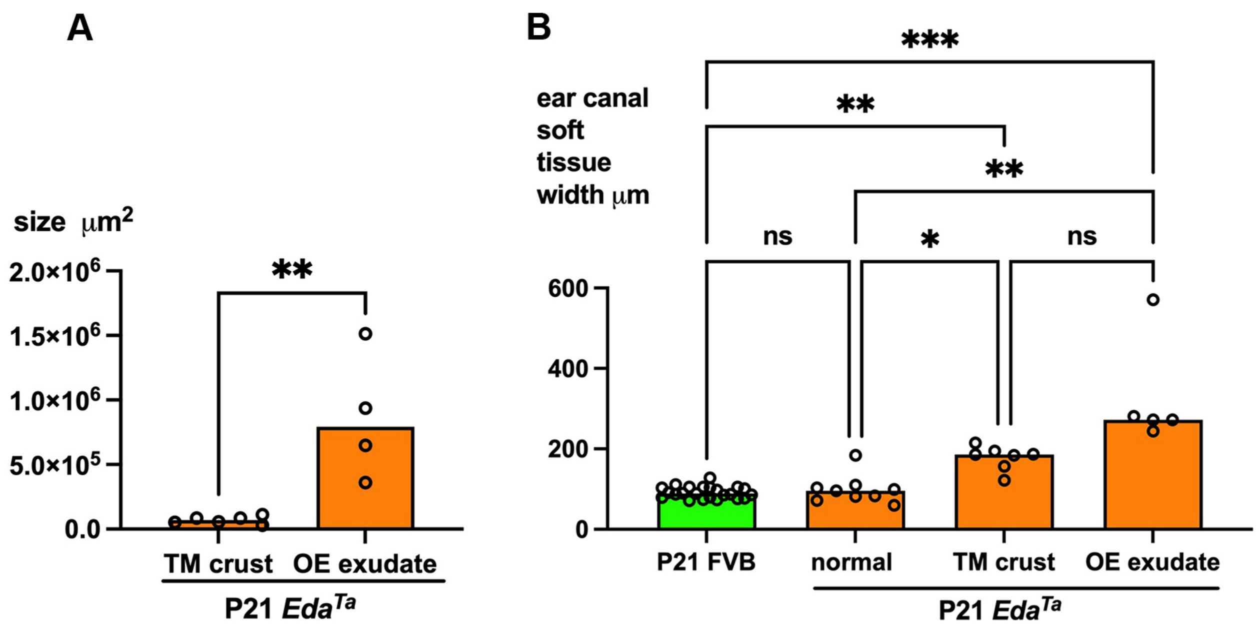

Fig. S1. Morphometric analysis of tympanic membrane crusts and otitis externa exudate, and inflammatory thickening of ear canal soft tissue in P21 *Eda Ta* mice.

(A) Tympanic membrane crusts have similar composition to otitis externa exudate but are <10% the size of exudate accumulations.

(B) P21 *Eda Ta* ear canals can be categorised as normal healthy, those with tympanic membrane crusts and those with otitis externa. The thickness of ear canal soft tissues is comparable in healthy *Eda Ta* ear canals and FVB controls, while *Eda Ta* ear canals with tympanic membrane crusts and otitis externa exudates are thickened.

Data are represented as points and the median as the histogram bar.

Data in graph (A) was analysed with a Mann-Whitney test, and in (B) using Kruskal-Wallis tests followed by Dunn's multiple comparison tests. Two-tailed tests: not significant ns P>0.05; *P<0.05*; **P<0.01; ****P*< 0.001; *****P*< 0.0001.

Disease Models & Mechanisms • Supplementary information

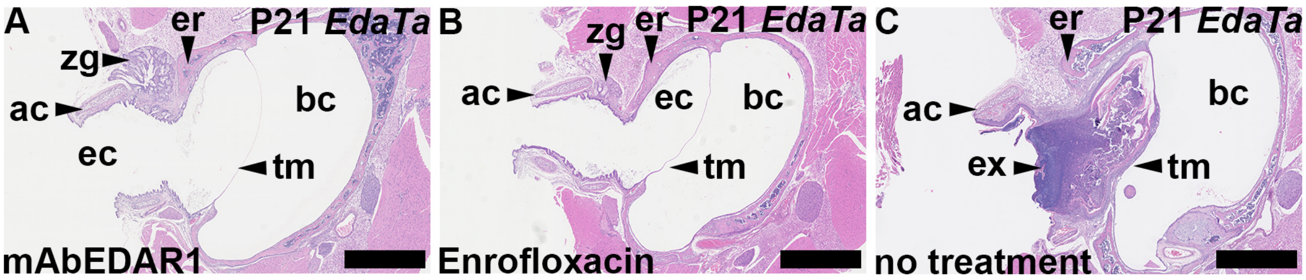

Fig. S2. Otitis externa is absent in P21 *Eda Ta* mice treated with anti-EDAR antibody (mAbEDAR1) or Enrofloxacin.

(A) The Zymbal's gland is rescued by prenatal treatment with mAbEDAR1 and there is no otitis externa.
(B) The Zymbal's gland is small in an Enrofloxacin treated mouse but there is no otitis externa.
(C) An example of an untreated P21 *Eda Ta* mouse with otitis externa, the ear canal is filled with suppurative exudate mixed with squamous cells. The Zymbal's gland is not evident in its normal location between the annular cartilage and ectotympanic ring. ac, annular cartilage ; be, bulla cavity; ec, ear canal; er, ectotympanic ring; ex, inflammatory exudate; tm, tympanic membrane ; zg, Zymbal's gland. Scale Bars: (A-C) 1 mm.

Disease Models & Mechanisms • Supplementary information

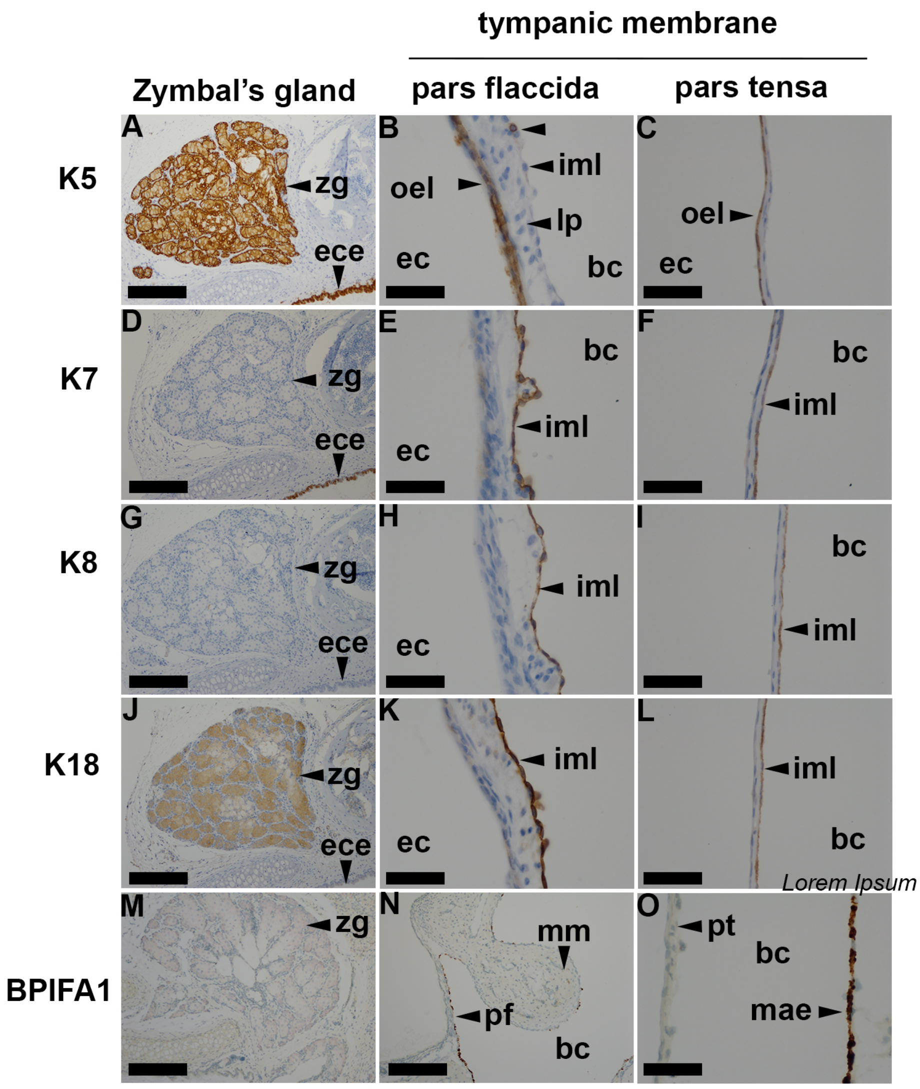

Fig. S3. Tympanic membrane and Zymbal's gland cytokeratin and BPIFA1 expression.

Tissue sections (A-L) P22 *MecomJbo/+,* (M-0) P21 *Fbxo11Jf/+* are from mouse strains that do not have otitis externa. Note BPIFA1-positive mesenchyme associated epithelium in P21 *Fbxo11Jfl+* with a bulla cavitation defect.

(A-C) Zymbal 's gland, and ear canal stratified squamous epithelium and outer epithelium of the pars flaccida and pars tensa have high populations of KS-positive cells, whereas the epithelium of the inner mucosal layer epithelium has only scattered KS-positive cells (unlabelled arrowhead). (D-L) K7-, K8- and K18-positive cells are present in inner mucosal layer epithelium of the pars flaccida and pars tensa ; K18 stains the Zymbal's gland. K7 stains the ear canal epithelium. (M-0) *In situ* hybridization signals for BPIFA 1 are restricted to epithelium of the inner mucosal layer of the pars flaccida and that covering the manubrium of the malleus but not the pars tensa. be, bulla cavity; ec, ear canal; ece, ear canal epithelium; iml, inner mucosal layer epithelium; lp, lamina propria; mae, mesenchyme associated epithelium; mm, manubrium of the malleus; oel, outer epithelial layer; pf, pars flaccida ; pt, pars tensa ; zg, Zymbal's gland.

Scale Bars: (A,D,G,J,M,N) 200 µm; (B,C,E,F,H,I,K,L,O) 50 µm.

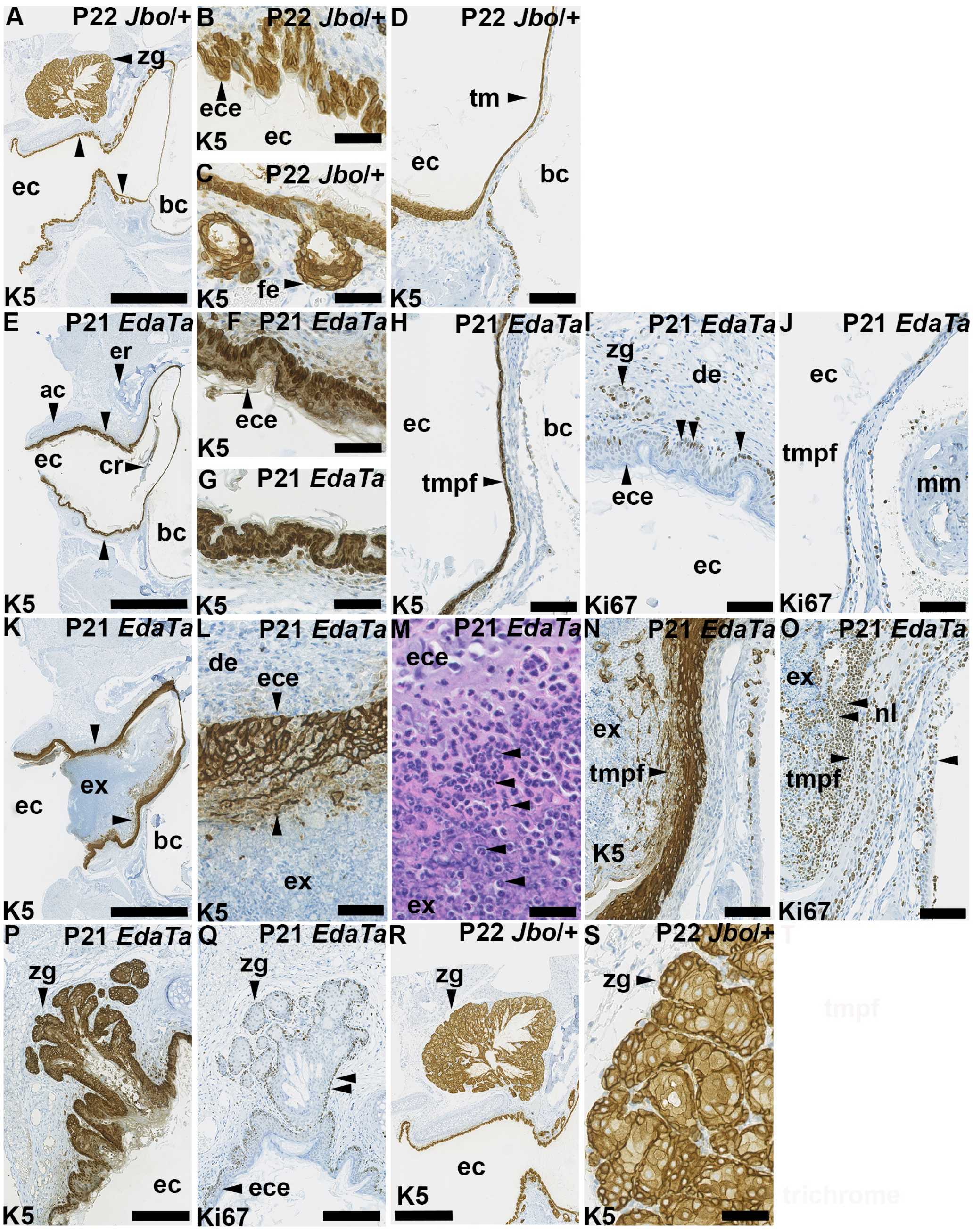

Fig. S4. Epithelial hyperkeratosis and inflammation in P21 *Eda Ta* mice with otitis externa.

(A-D) P22 *MecomJbol*+ ear canal.
(E-J) P21 *Eda Ta* ear canal with tympanic membrane crust.
(K-0) P21 *Eda Ta* ear canal with suppurative otitis externa.
(P,Q) P21 *Eda Ta* Zymbal's gland and (R,S) P22 *MecomJbol*+ Zymbal's gland.
(A-D) P22 *MecomJbol*+ healthy ear canal with normal epithelial lining.
Images A-D are of the same ear canal at different magnifications.
(B,C) Ear canal epithelium from the sites indicated by the unlabelled vertical arrowheads in low power image A.
(B) Non-haired skin overlying the annular cartilage and (C) haired skin of the osseous canal have KS-positive basal and suprabasal epithelial cells, but the acellular superficial squames are negative.
(D) The outer epithelial layer of the tympanic membrane is KS-positive.
(E-J) P21 *Eda Ta* ear canal with tympanic membrane crust. Ear canal epithelium from the sites indicated by the unlabelled vertical arrowheads in low power image E.
(F) Non-haired skin between the annular cartilage and ectotympanic ring and (G) non-haired stratified squamous epithelium of the caudal surface of osseous canal has KS-positive basal and suprabasal cells, but the acellular superficial squames are negative. (H) The outer epithelial layer stains of the tympanic membrane is KS-positive.

(I) Ki67 staining of recently proliferated basal cells in ear canal epithelium and Zymbal 's gland (arrowheads) , and in the tympanic membrane outer epithelial layer and scattered inflammatory cells in the bulla cavity lumen.

Disease Models & Mechanisms • Supplementary information

(K-O) P21 *Eda Ta* ear canal with suppurative otitis externa, (K-L) are low and high power images of same ear canal. (L,N) Non-haired hyperkeratotic skin between the annular cartilage and ectotympanic ring is KS-positive and has a porous appearance and (M) shows intraepithelial and exudate polymorphonuclear neutrophil leukocytes (arrowheads).
(N) The outer epithelial layer of the hyperkeratotic tympanic membrane pars flaccida is KS-positive, and has a porous appearance due to infiltrating neutrophils. (0) Ki67 staining shows the neutrophils are recently proliferated (opposing arrowheads indicate margins of the tympanic membrane).
(P,Q) The hypoplastic P21 *Eda Ta* Zymbal's gland is uniformly KS-positive and has Ki67-positive basal cells (Q). (R,S) Normal Zymbal's glands in a P22 *MecomJbol*+ mouse in which basal and suprabasal sebocytes have cytoplasmic K5 staining.
ac, annular cartilage ; be, bulla cavity; er, tympanic membrane crust; de, dermis ; ec, ear canal; ece, ear canal epithelium; er, ectotympanic ring; ex, exudate; fe, external follicle root sheath epithelium; nl, neutrophil leukocyte; tm, tympanic membrane; tmpf, tympanic membrane pars flaccida ; zg, Zymbal's gland.
Scale Bars: (A,E,K) 1 mm; (R) 500 µm; (P,Q) 250 µm; (D,H,I,J,N,O) 100 µm. (B,C,F,G,L,S) 50 µm, (M) 25 µm.

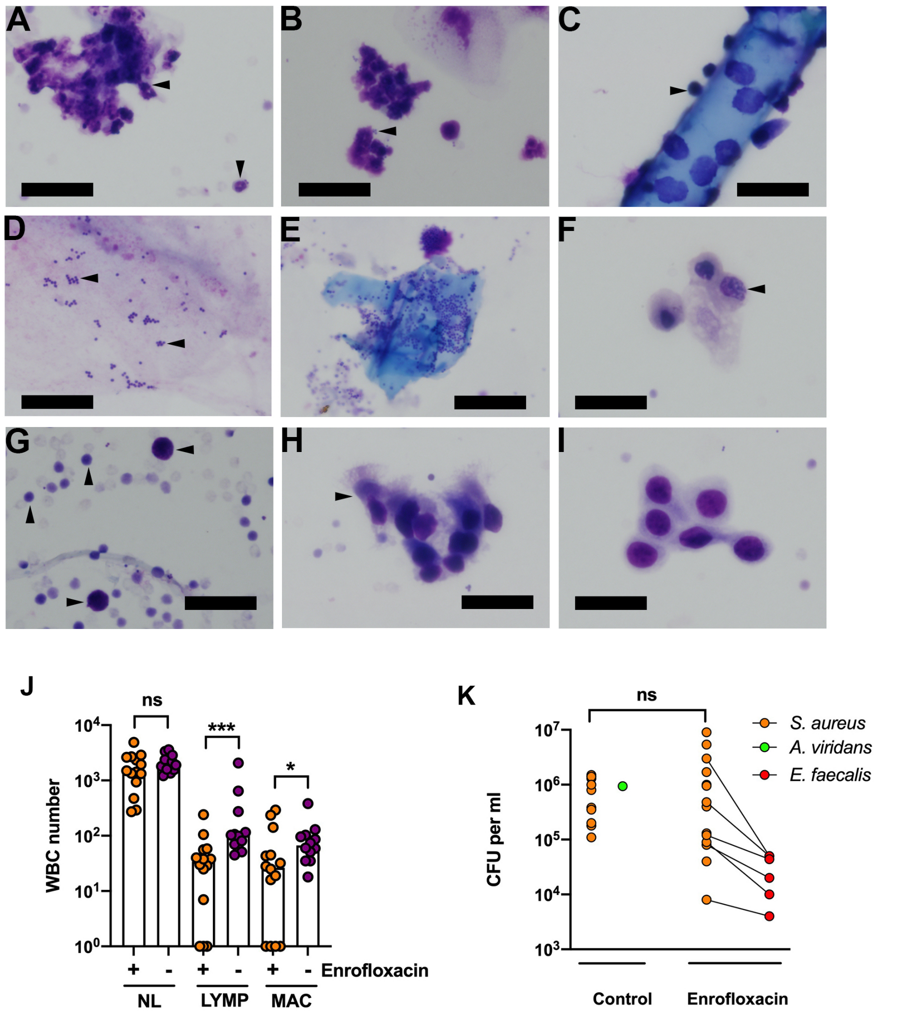

Fig. S5. Nasal cytology and microbiology of P21 *Eda Ta* mice treated with Enrofloxacin.

(A-I) White blood cells (WBC) and epithelial cells in nasal wash samples.
(A) Neutrophils , solitary viable cell (vertical arrowhead) and cluster of degenerate cells (horizontal arrowhead); (B) polymorphonuclear neutrophils engaged in phagocytosis of cocci; (C) neutrophils adherent to a fibre ; (D) cocci in mucus; (E) cocci adherent to a squamous cell; (F) macrophage with a reniform nucleus and cytoplasmic vacuoles ;
(G) lymphocytes (horizontal arrowheads) and (smaller) red blood cells (vertical arrowheads);
(H) columnar ciliated epithelial cells; (I) cohesive cluster of basal epithelial cells. Giemsa stained cytospin preparations. Scale bars 20 µm.
(J) WBC numbers and (K) bacterial isolates in nasal washes of control and Enrofloxacin treated mice. Data are represented as points. In panel J, the histogram bar represents the median.
NL, neutrophils; LYMP, lymphocytes; MAC, macrophages. Note zero leukocyte counts are given a nominal value of 1 to graph on a log scale. In panel K, lines link nasal samples in which *E. faecalis* is co-cultured with S. *aureus.*
Data were analysed with Mann-Whitney tests.
Two-tailed tests: not significant ns *P*>0.05 ; **P*<0.05* ; ****P*<0.001.

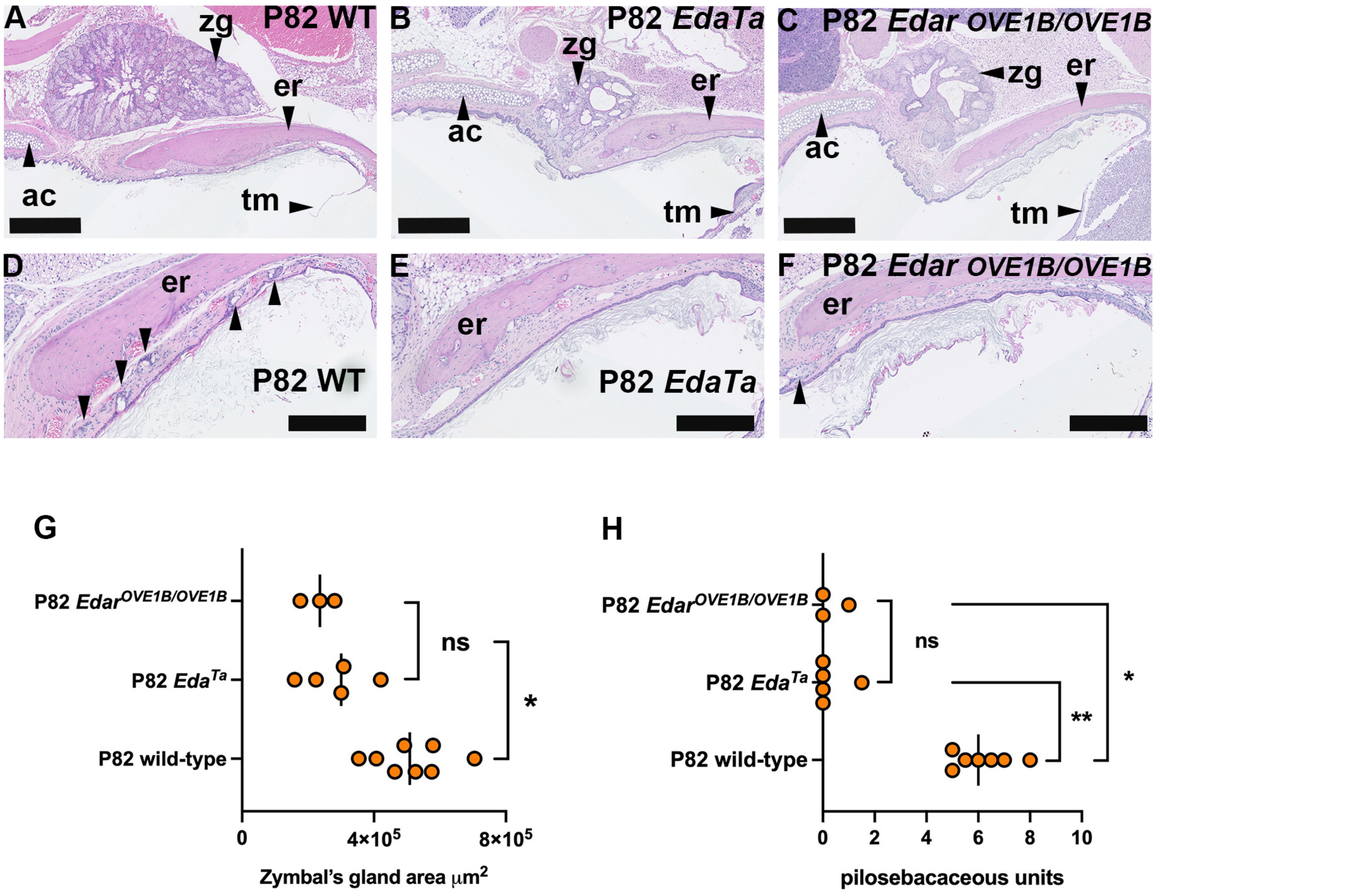

Fig. S6. *Edar* deficient mice have Zymbal's gland hypoplasia and ear canal hypotrichosis.
(A-C) Examples of Zymbal's glands from (A) P82 wild-type, (B) *P82 Eda Ta* and (C) P82 Edar deficient mice (*EdarOVE1B/OVE1B*) (H&E stain).
(D-F) Pilosebaceous units (arrowheads) in ear canal skin of (D) P82 wild-type and (F) P82 *EdarOVE1BIOVE1B* mice; (E) P82 *Eda Ta* ear canal has no pilosebaceous units at this section level.
(G) The size of the Zymbal 's gland at P82 is not significantly different in *EdarOVE1BIOVE1B* and *Eda Ta* mice and both are significantly smaller than same aged wild-type mice.
(H) The ear canals of *EdarOVE1BIOVE1B* and *Eda Ta* mice have a low density of pilosebaceous units compared with wild-type mice.
Data in graphs are represented as points and the bar is the median value. Two-tailed Kruskal-Wallis test and Dunn's multiple comparison test; not significant ns $P>0.05$; *$P<0.05$; **$P<0.01$.
ac, annular cartilage ; er, ectotympanic ring; tm, tympanic membrane ; zg, Zymbal's gland. Scale Bars: (A-C) 500 µm, (D-F) 250 µm.

Disease Models & Mechanisms • Supplementary information

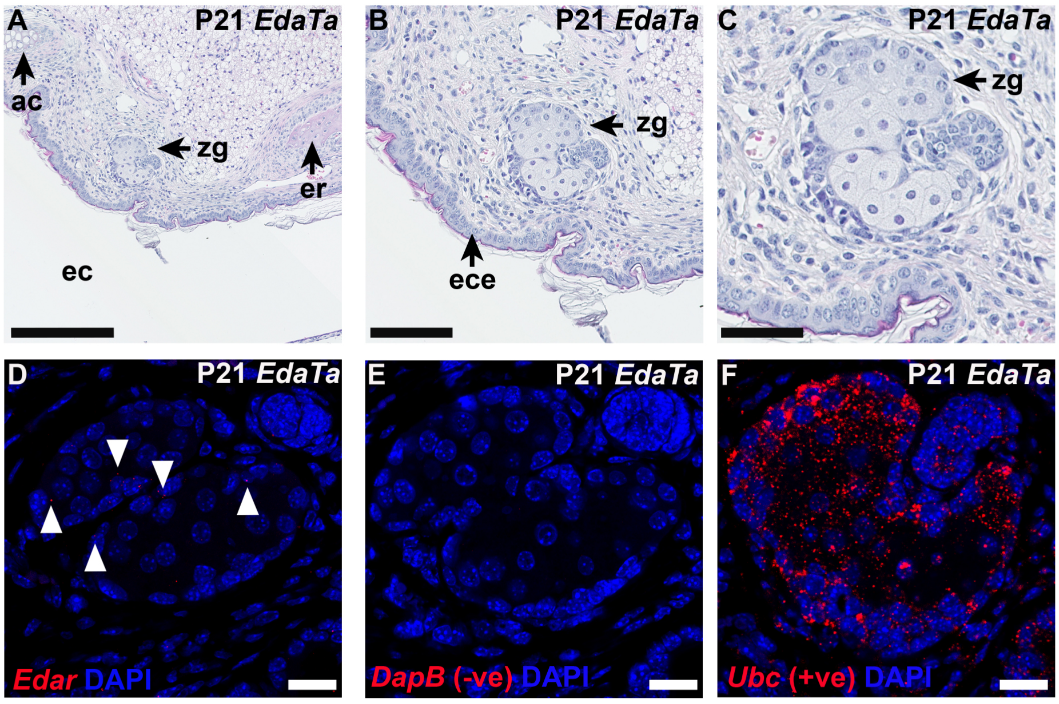

Fig. S7. *Edar* expression in P21 *EdaTa* Zymbal's gland.
(A-C) Different magnifications of the same P21 *EdaTa* Zymbal's gland section (H&E stain). (D-F) Nearby serial sections of Zymbal's gland are fluorescent ISH preparations with DAPI nuclear counter stain.

(D) *Edar* signals are punctate spots (arrowheads).
(E) ISH signals are absent with the negative control probe (*DapB*).
(F) The positive control probe (*Ubc*) shows intense ISH signals.
The ISH images (D-F) (and those of Figure 5) were all acquired using the same microscope settings. ac, annular cartilage; ec, ear canal; ece, ear canal epithelium; er, ectotympanic ring; zg, Zymbal's gland.
Scale Bars: (A) 250 µm; (B) 100 µm; (C) 50 µm; (D-F) 20 µm.

Disease Models & Mechanisms • Supplementary information

Table S1. Reduction in otitis externa and otitis media prevalence in agonist anti-EDAR antibody (mAbEDAR1) treated and in Enrofloxacin treated P21 EdaTa mice. The prevalence of otitis externa and otitis media in treatment groups was compared to untreated controls using Fisher's exact tests. Note that this is done for assessments on a per mouse basis (where affected mice have either unilateral or bilateral disease) or on a per ear basis. Otitis externa and otitis media are significantly associated in individual ears of untreated P21 EdaTa mice. The association was analysed with a Fisher's exact test. Statistically significant results *P*<0.05 are highlighted in bold.

	OTITIS EXTERNA			
treatment	Number of mice	not affected	unilateral	bilateral
untreated	12	6	6	0
Enrofloxacin	14	14	0	0
mAbEDAR1	8	8	0	0

	OTITIS EXTERNA PER MOUSE		
treatment	not affected	affected	*P*
untreated	6	6	
Enrofloxacin	14	0	**0.004**
mAbEDAR1	8	0	**0.0419**

	OTITIS EXTERNA PER EAR		
treatment	not affected	affected	*P*
untreated	18	6	
Enrofloxacin	28	0	**0.0066**
mAbEDAR1	16	0	0.0645

	OTITIS MEDIA			
treatment	Number of mice	not affected	unilateral	bilateral
untreated	12	4	7	1
Enrofloxacin	14	12	0	2
mAbEDAR1	8	8	0	0

	OTITIS MEDIA PER MOUSE		
treatment	not affected	affected	*P*
untreated	4	8	
Enrofloxacin	12	2	**0.0138**
mAbEDAR1	8	0	**0.0047**

	OTITIS MEDIA PER EAR		
treatment	not affected	affected	*P*
untreated	15	9	
Enrofloxacin	24	4	0.1063
mAbEDAR1	16	0	**0.006**

		OTITIS MEDIA		
		absent	present	*P*
OTITIS EXTERNA	absent	14	4	**0.0147**
	present	1	5	
